# A hemoglobin-based nanozyme with ruthenium-induced nanomotor ability exhibiting photothermal and chemodynamic responses

**DOI:** 10.1007/s00604-026-08176-3

**Published:** 2026-06-10

**Authors:** Gökçe Çoban, Esin Akbay Çetin, İrem Yağmur Gök, Neşen Betül Kaya, Burcu Gökçal Kapucu, M. Ali Onur, Mustafa Polat, Çiğdem Kip, Ali Tuncel

**Affiliations:** 1 Chemical Engineering Department, Hacettepe University, Ankara, 06800 Turkey; 2 Graduate School of Science & Engineering, Hacettepe University, Ankara, 06800 Turkey; 3 Department of Biology, Hacettepe University, Ankara, 06800 Turkey; 4 Division of Bioengineering, Graduate School of Science & Engineering, Hacettepe University, Ankara, 06800 Turkey; 5 Department of Physics Engineering, Hacettepe University, Ankara, 06800 Turkey

**Keywords:** Hemoglobin, Ruthenium, Nanozyme, Glioblastoma, Chemodynamic therapy

## Abstract

Ruthenium functionalized organometallic nanoparticles (Hb@Ru NPs) ca. 10 nm in size, containing crystalline Ru phases were synthesized by a new, single-stage hydrothermal protocol using hemoglobin (Hb) as the skeleton. Compared to currently reported nanozymes, Ru@Hb NPs demonstrated enhanced catalase- and and peroxidase-mimicking activities, produced superoxide (O_2_^•-^) and singlet oxygen (^1^O_2_) radicals and showed significant glutathione depletion. The maximum substrate consumption rates of 107.5 mM mg^-1^s^-1^ and 6.94 µM mg^-1^s^-1^, were obtained for catalase-like and peroxidase-like activities, respectively. Hb@Ru NPs exhibited ruthenium induced nanomotor behavior which was utilized to enhance the interaction between tumor cells and nanozyme. Photothermal conversion behavior of Hb@Ru NPs was demonstrated with the temperature elevations of up to 29 °C under NIR laser irradiation. Therapeutic potential of Hb@Ru NPs was evaluated using T98G glioblastoma and HepG2 cells. In-vitro combinatorial photothermal/chemodynamic therapy (PTT&CDT) with T98G cells achieved up to 92.7% cell death, with effective intracellular ROS formation and yielded an apoptotic rate of 63.32%, as determined by flow cytometry. TUNEL staining demonstrated that Hb@Ru NPs significantly induced DNA fragmentation in T98G cells by combined PTT&CDT via producing the most pronounced apoptotic response. PTT&CDT with Hb@Ru NPs also suppressed the migration and proliferation of glioblastoma cells, significantly inhibiting the wound closure in stratch assay.

**Graphical Abstract:**

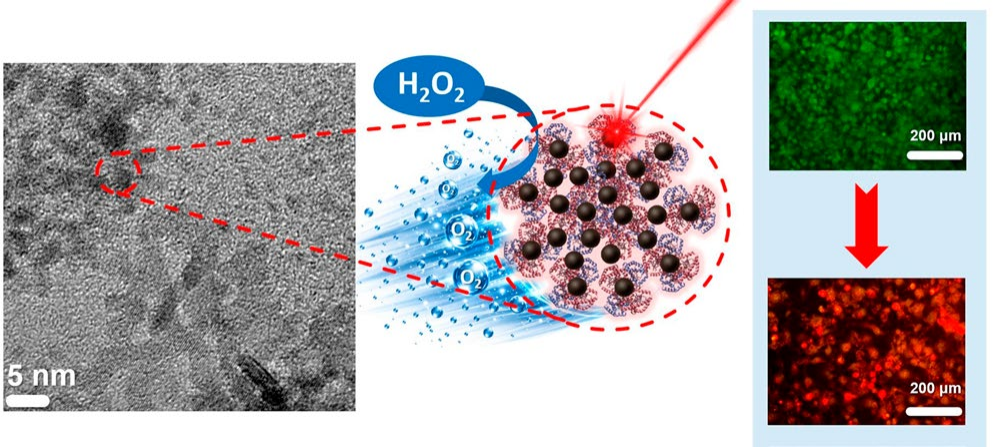

**Supplementary Information:**

The online version contains supplementary material available at 10.1007/s00604-026-08176-3.

## Introduction

The nanomaterials with intrinsic enzyme-like property (nanozymes) have attracted significant attention as promising sensing platforms. A smartphone-assisted colorimetric detection of glucose was performed using molecularly imprinted nanozymes [1]. A molecularly imprinted nanozyme having artificial substrate recognition was evaluated for colorimetric sensing of levodopa [2]. The nanozymes also play a prominent role in the synergistic antibacterial and antitumoral therapy applications including chemodynamic function. However, the clinical application of chemodynamic therapy (CDT) alone is usually restricted due to low catalytic efficiency and hypoxic tumor microenvironment which can limit generation of ROS [3]. The co-treatment of CDT and photothermal therapy (PTT) in an integrated nanoplatform is considered as a good strategy to induce synergistic therapeutic efficiency by increasing catalytic reactions and promoting the generation of ROS [4]. In recent years, promising approaches were designed for synthesizing and evaluating high-performance PTT agents. Biocompatible photo- and thermoresponsive crosslinked single-chain NPs was proposed for PTT with high photothermal heating performance [5]. The yeast cells including the intercellular bio-photothermal agent was evaluated with a high photothermal conversion efficiency [6]. Genetically modified bacteria were used to produce Ag_2_S NPs under ecofriendly conditions for PTT and photoacoustic imaging [7].

Owing to its intrinsic biocompatibility and biodegradability, as well as presence of redox activity, Hb is a promising metalloprotein for CDT systems. The coordination of Hb with suitable metal ions can promote its enzyme-like activity and afford strong photothermal properties [8]. Hb-PDA-GOx nanohybrids exhibited high ROS production and favorable photothermal conversion efficiency under NIR laser irradiation [9]. Hb-decorated boron-carbon nanosheets was synthesized for photoacoustic (PA) imaging-guided combinatorial PTT&CDT [10]. Recently, a nanofiber-based drug release device was developed by doping platinum pro-drugs and Hb into mesoporous silica nanoparticles immobilized within nanofibers for glioblastoma therapy [11]. However, the synthesis of Hb containing therapeutic agents mostly involve multiple steps over extended durations also by including template materials in some cases [8–13]. On the other hand, there has been an increasing interest on Ru functionalized therapy agents in the form of bimetallic nanoalloys or organometallic nanoplatforms [14–18]. However, limited number of studies were made on the therapy agents synthesized by combination of Hb with a Ru precursor. An intelligent core–shell microneedle patch in the form of MXene@RuO_2_ heterojunction with glucose oxidase and Hb was proposed for ICB therapy [12]. A nanoplatform capable of releasing mitochondria-targeting ruthenium sonosensitizer was proposed for triad chemo-sonodynamic immunotherapy [19]. Multi-stage strategies were also followed for the synthesis of these synergistic therapy agents containing Hb and Ru species [14–19]. In this work, a new synergistic nanoplatform in the form of ruthenium-induced nanomotor ability and endowed with superior enyzme-mimetic activities (hemoglobin-ruthenium nanoparticles, Hb@Ru NPs) was directly synthesized via a facile, single-stage hydrothermal protocol using Hb as the organic component and Ru as the metal center. The combination of Ru together with denaturated Hb provided a synergistic therapy agent superior enzyme-mimetic activities and superior ROS generation ability. The nanoplatform synthesized was evaluated for synergistic PTT&CDT of glioblastoma and liver cancer cells, through in-vitro studies, without any structural modification. Here, we wish to present the synthesis route of Hb@Ru NPs, its chemical and morphological characteristics and to demonstrate its potential for the synergistic tumor therapy.

## Results and discussion

### Characterization of Hb@Ru NPs

**Fig. 1:**
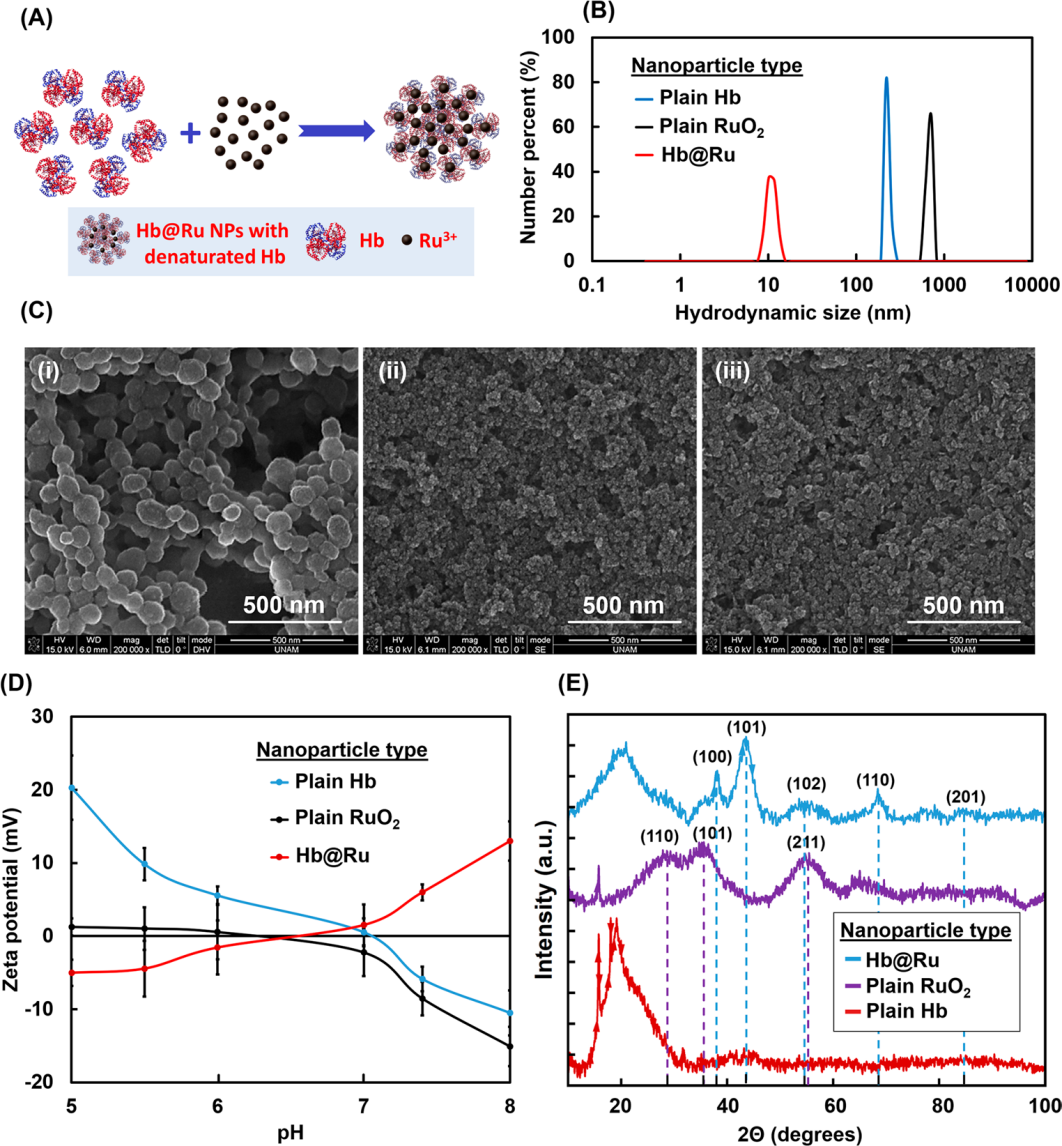
**A** Schematic view of chemical route used for synthesis of Hb@Ru NPs. **B** Hydrodynamic size distribution curves of plain Hb, plain RuO_2_ and Hb@Ru NPs obtained by Dynamic Light Scattering, Medium: DMEM, pH 7.4, (**C**) SEM images of plain Hb, Plain RuO_2_ and Hb@Ru NPs. Magnification: X200.000, Scale-bar: 5000 nm, (**D**) The variation of zeta-potential of plain Hb, plain RuO_2_ and Hb@Ru NPs with pH, (**E**) X-ray diffraction spectra of plain Hb, plain RuO_2_ and Hb@Ru NPs

The synthesis of Hb@Ru NPs was performed at 140 °C using ruthenium (III) chloride hydrate as the metal precursor and Hb as the protein-based skeleton. ICP-MS determination provided 40% w/w Ru content in Hb@Ru NPs (Fig. 1A). Thermogravimetric analysis demonstrated that the weight loss of dry Hb@Ru NPs was 37.7% when the temperature was increased up to 800 °C (Figure S1). A certain extent of carbon-based material may remain in the pyrolyzed sample obtained at 800 °C. When TGA and ICP-MS results are assessed together, one can conclude that half of Hb@Ru NPs roughly consists of an organic component based on denaturated Hb while the other half comprises ruthenium (Ru).

The hydrodynamic size distribution curves of Hb@Ru NPs and plain Hb NPs synthesized in the absence of Ru salt and plain (hydrous) RuO_2_ NPs synthesized in the absence of Hb via the same hydrothermal protocol are given in Fig. 1B. The mode hydrodynamic size values for plain Hb NPs and plain RuO_2_ NPs were determined as 227 nm and 676 nm, respectively. However, the mode hydrodynamic size of Hb@Ru NPs was found as ca. 10 nm (Fig. 1B). The SEM images of plain Hb, plain RuO_2_ and Hb@Ru NPs are presented in Fig. 1C. The mode particle size of plain Hb NPs was determined as 87 nm by the image analysis of SEM photo in Fig. 1C(i). The mode hydrodynamic size of Hb NPs measured by DLS (i.e. 277 nm) can be explained by swelling or limited micro-agglomeration of plain Hb NPs. However, the mode hydrodynamic size of RuO_2_ NPs determined by DLS is much higher (i.e. 667 nm) with respect to the mode size of individual RuO_2_ NPs in the SEM image (i.e. ca. 10 nm) (Figure 1C(ii)). Despite a dense ultrasonication was applied, plain RuO_2_ NPs could be dispersed only in the form of micro-agglomerates in the aqueous cell culturing medium. However, the mode hydrodynamic size of Hb@Ru NPs measured by DLS in DMEM (i.e. 10 nm) was roughly consistent with the mode size of individual Hb@Ru NPs in the SEM image (Figure 1C(iii)). One can conclude that Hb likely acts as an effective stabilizer in the synthesis of Hb@Ru NPs, enabling the formation of colloidally stable NPs resistant to agglomeration in aqueous media. The hydrothermal protocol applied by the combination of Ru salt with Hb produced ultrasmall Hb@Ru NPs with a hybrid organometallic structure well-stabilized by denaturated Hb.

The variation of zeta potential with pH is presented in Fig. 1D for plain Hb, plain RuO_2_ and Hb@Ru NPs. Hb@Ru NPs had an appreciable surface charge in the studied pH range, likely originated from the amino acid segments of denatured Hb. Plain Hb NPs exhibit positive charge at pH 5–6 due to the protonation of amino groups and negative charge at pH ≥ 7.5 depending on the deprotonation of carboxyl groups of amino acid units. As expected, plain RuO_2_ NPs are almost neutral (i.e. with very low surface charge) up to pH 7.0. The behavior in Fig. 1D supports the hypothesis on the stabilizer role of denaturated Hb in the formation of ultrasmall Hb@Ru NPs.

The crystal planes of Ru (100), Ru (101), Ru (102), Ru (110), Ru (201) are observed in the XRD spectra of Hb@Ru NPs (JCPDS card no: 04-018-8034) (Fig. 1E). The structural parameters calculated based on the XRD spectra of Hb@Ru NPs are given in Table S1 of the Supporting Information. The average values of crystallite size, crystal volume and d-spacing of Hb@Ru NPs were calculated as 4.7 nm, 0.0874 nm and 0.17262 nm, respectively. Plain hydrous RuO_2_ NPs with a tetragonal crystalline structure was obtained by the hydrothermal treatment of ruthenium (III) chloride hydrate in an aqueous medium not containing Hb at 140 °C (Fig. 1D) [20]. As anticipated plain Hb NPs exhibited an amorphous structure (Fig. 1E).

**Fig. 2:**
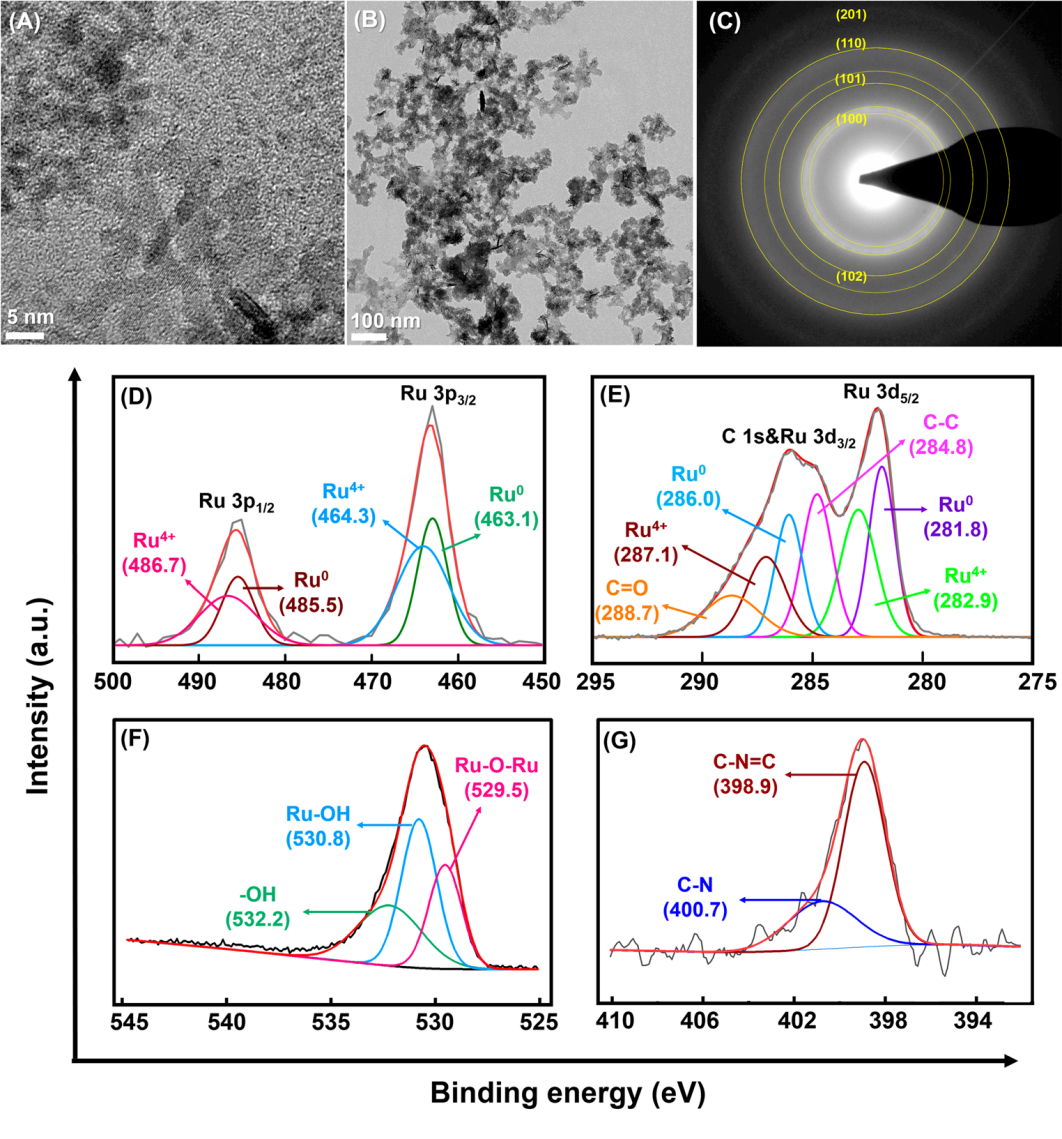
**A** HRTEM image showing the d-spacing values in Hb@Ru NPs at different regions. **B** TEM image of Hb@Ru NPs. The scale bars are shown on the images, (**C**) SAED pattern of Hb@Ru NPs. Core level XPS spectra for (**D**) Ru 3p (**E**) Ru 3d, (**F**) O 1s, and (**G**) N 1s scan of Hb@Ru NPs

TEM images of Hb@Ru NPs are given in Fig. 2A and B. The nanoparticles smaller than 10 nm are clearly observed in the HRTEM image in Fig. 2A. The mean hydrodynamic size of ca. 10 nm obtained in DLS can be explained by the limited micro-agglomeration of individual Hb@Ru NPs. The zoomed-in HRTEM images obtained from different regions of the HRTEM micrograph are included in Figure S2. The d-spacing values between 0.109 and 0.162 nm, measured on the image are in the range given for d-spacing values calculated for different planes of crystalline Ru phases (Table S1). The crystal planes of Ru (100), Ru (101), Ru (102), Ru (110) and Ru (201) are clearly observed in the SAED pattern of Hb@Ru NPs (Fig. 2C). All these findings confirmed the presence of crystalline Ru phases within Hb@Ru NPs.

The survey X-ray photoelectron spectroscopy of Hb@Ru NPs is given in Figure S3 of the Supporting Information. The peaks belonging to Ru 3d5, Ru 3d3, C 1s, N 1s, Ru 3p1, Ru 3p3 and O 1s peaks are observed. The peaks assigned to Ru 3p3/2 and Ru 3p1/2 electron levels were observed in the core level spectra for Ru 3p scan (Fig. 2D). The deconvoluted peaks assigned to Ru^0^ and Ru^4+^ valence states were obtained at 463.1 and 464.3 eV, respectively within the peak belonging to Ru 3p3/2 electron level. The peaks assigned to Ru^0^ and Ru^4+^ valence states were also obtained at 485.5 and 486.7 eV within the peak belonging to Ru 3p1/2 electron level (Fig. 2D). The energy separation between Ru 3p3/2 and 3p1/2 peaks is approximately 22.4 eV for both oxidation states, which is in good agreement with the characteristic spin-orbit splitting of Ru, confirming the reliability of peak assignment. These results indicate the coexistence of metallic and oxidized Ru species on the surface.

The peaks belonging to Ru 3d5/2 and Ru 3d3/2 electron levels are found in the core level spectra for Ru 3d scan (Fig. 2E). The peaks at 281.8 eV and 282.9 eV are identified by the deconvolution of Ru 3d5/2 level and assigned to Ru^0^ and Ru^4+^ valence states, respectively. The deconvolution of the peak belonging to Ru 3d3/2 level is more complex since it also contains peaks belonging to C 1s level [21]. The peaks at 286.0 eV and 287.1 are assigned to Ru^0^ and Ru^4+^ valence states, respectively, by the deconvolution of the peak belonging to Ru 3d3/2 level (Fig. 2E). In addition, the binding energy separation between the spin-orbit split components (Ru 3d5/2 and Ru 3d3/2) were constrained to ~ 4.2 eV during the fitting procedure. The presence of C 1s peaks within the deconvoluted core level spectra for Ru 3d scan is evidence for organic species found on the surface of Hb@Ru NPs. Each heme group of Hb contains a porphyrin ring [22]. The deconvoluted peaks in the core level spectra for Ru 3d scan at 284.8 eV and 288.7 eV are assigned to C-C and C = O groups, respectively. Hence, the presence of organic segments coming from the denatured Hb on Hb@Ru NPs was shown since C-C and C = O groups are found in the porphyrin rings and amino acids of the Hb molecule [22].

The peaks assigned to Ru-O-Ru, Ru-OH and OH groups were obtained at 532.2 eV, by deconvolution of O 1s scan (Fig. 2F). In N 1s scan, the peaks at 398.9 eV and 400.7 eV are assigned to C-N = C and C-N groups, respectively which are also found in Hb (Fig. 2G). The XPS characteristics of Hb@Ru NPs are summarized in Table S2.

### Nanozyme function of Hb@Ru NPs

**Fig. 3:**
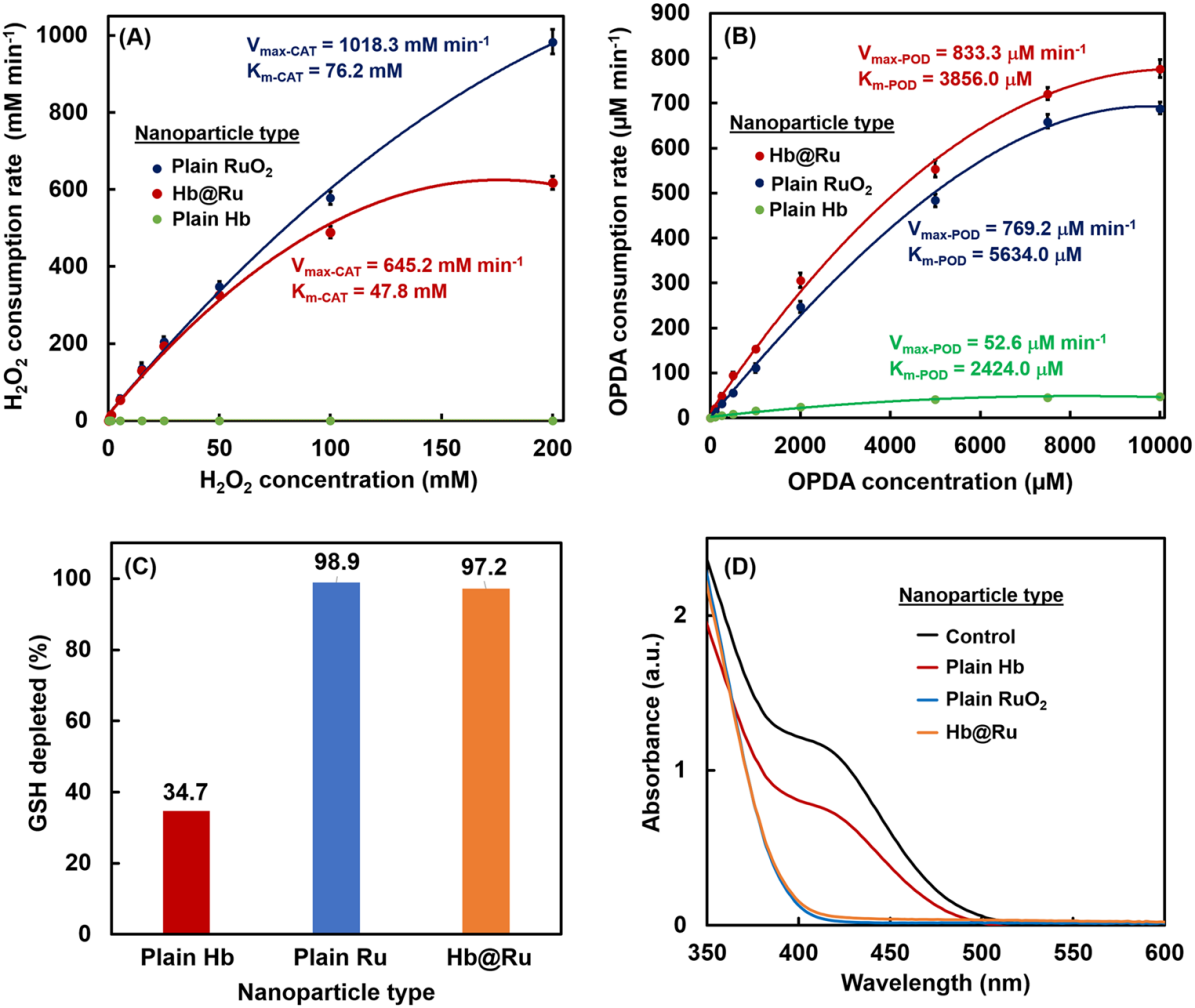
Michaelis-Menten plots for (**A**) CAT-like activity and (**B**) POD-like activity of plain Hb, plain RuO_2_ and Hb@Ru NPs. Nanozyme concentration: 0.05 mg mL^-1^ for CAT-like activity and 1.0 mg mL^-1^ for POD-like activity. Medium: 1x PBS buffer at pH 7.4. Temperature: 37 °C, 300 rpm, (**C**) The comparison of GSH depleted by plain Hb, plain RuO_2_ and Hb@Ru NPs. (**D)** Sample UV-Vis spectra recorded for GSH depletion with plain Hb, plain RuO_2_ and Hb@Ru NPs. Nanozyme concentration: 1.0 mg mL^-1^. Medium: 1x PBS buffer at pH 7.4, Temperature: 37 °C. Initial GSH concentration: 0.5 mM, Time: 1 h

Hb@Ru NPs behaved as a multifunctional nanozyme with catalase-like (CAT-like) and peroxidase-like (POD-like) activity. Michaelis-Menten Plots for CAT-like activities of plain Hb, plain RuO_2_ and Hb@Ru NPs in 1x PBS buffer at 37 °C as a representative medium for serum and extracellular fluids in terms of pH and ionic composition are given in Fig. 3A. The related Lineweaver-Burk plots are included in Figure S4A. The highest CAT-like activity was obtained with plain RuO_2_ NPs while plain Hb NPs did not exhibit any CAT-like activity. The CAT-like activity of Hb@Ru NPs was slightly lower with respect to plain RuO_2_ NPs. Hence, Ru is the key component enhancing the CAT-like activity of Hb@Ru NPs. The CAT-like activity almost linearly increased with the increasing concentration of Hb@Ru NPs (Figure S5A). The rising of temperature from 37 to 45 °C caused an appreciable increase in the CAT-like activity of Hb@Ru NPs (Figure S5B). Hence, a considerable increase should occur in CAT-like activity of Hb@Ru NPs by the temperature elevation that could be achieved under PTT conditions.

Michaelis-Menten plots for POD-like activities of plain Hb, plain RuO_2_ and Hb@Ru NPs in 1x PBS buffer at 37 °C are given in Fig. 3B. The highest POD-like activity was obtained with Hb@Ru NPs while plain Hb NPs gave the lowest POD-like activity. The POD-like activity of plain RuO_2_ NPs was slightly lower with respect to Hb@Ru NPs. The lower POD-like activity of plain RuO_2_ NPs can be explained by the formation of lower surface area due to the micro-agglomeration occuring in the aqueous medium (Fig. 2B). The POD-like activity increased with the increasing concentration of Hb@Ru NPs (Figure S5C). Elevating the temperature from 37 to 45 °C also increaed the POD-like activity of Hb@Ru NPs, suggesting a similar enhancement under PTT-induced heating conditions (Figure S5D).

The maximum substrate consumption rates for CAT-like and POD-like activities of Hb@Ru NPs were calculated as 107.5 mM mg^-1^ s^-1^ and 6.94 µM mg^-1^ s^-1^, respectively. These values are much higher with respect to the similar nanozyme-based synergistic therapy agents [23–25]. High CAT-like and POD-like activities are obtained with plain RuO_2_ NPs (Fig. 3A and B). Hence, the enhanced enzyme-mimetic activities of Hb@Ru NPs may be explained by the presence of well stabilized Ru species in its structure. The high surface area originated from the low mean size of Hb@Ru NPs (i.e. 10 nm) should be another factor that may provide superior enzyme-mimetic activity.

The comparison of GSH depleted by plain Hb, plain RuO_2_ and Hb@Ru NPs in 1x PBS buffer at 37 °C is given in Fig. 3C. The percent of GSH depleted was low with plain Hb NPs. The percentage values of GSH depletion by plain RuO_2_ NPs and Hb@Ru NPs were very close. Hence, Ru was again the key component controlling the rate of GSH depletion for plain RuO_2_ and Hb@Ru NPs. The related UV-Vis spectra obtained at a depletion period of 1 h with plain Hb, plain RuO_2_ and Hb@Ru NPs are included in Fig. 3D. The effect of Hb@Ru NP concentration on the GSH depletion in 1x PBS buffer at 37 °C is exemplified in Figure S6A together with the related spectra given in Figure S6B. Figure S6A shows that Hb@Ru NP concentration of 0.25 mg mL^-1^ is a critical value for almost complete depletion of GSH by Hb@Ru NPs.

The enzyme-mimetic activities and the GSH depletion behavior of Hb@Ru NPs obtained from an aqueous suspension (10 mg Hb@Ru NPs mL^-1^) stored in a refrigerator at +4 °C for approximately 5.5 months (exactly 171 days) were determined and compared with the initial values. The storage stability was also assessed by the determination of same parameters every 2 days after the storage period of 5.5 months. As seen in Figure S7, no significant change was observed in either enzyme-mimetic activities and GSH depletion ability of Hb@Ru NPs after the storage period.

### Nanomotor ability of Hb@Ru NPs

**Fig. 4:**
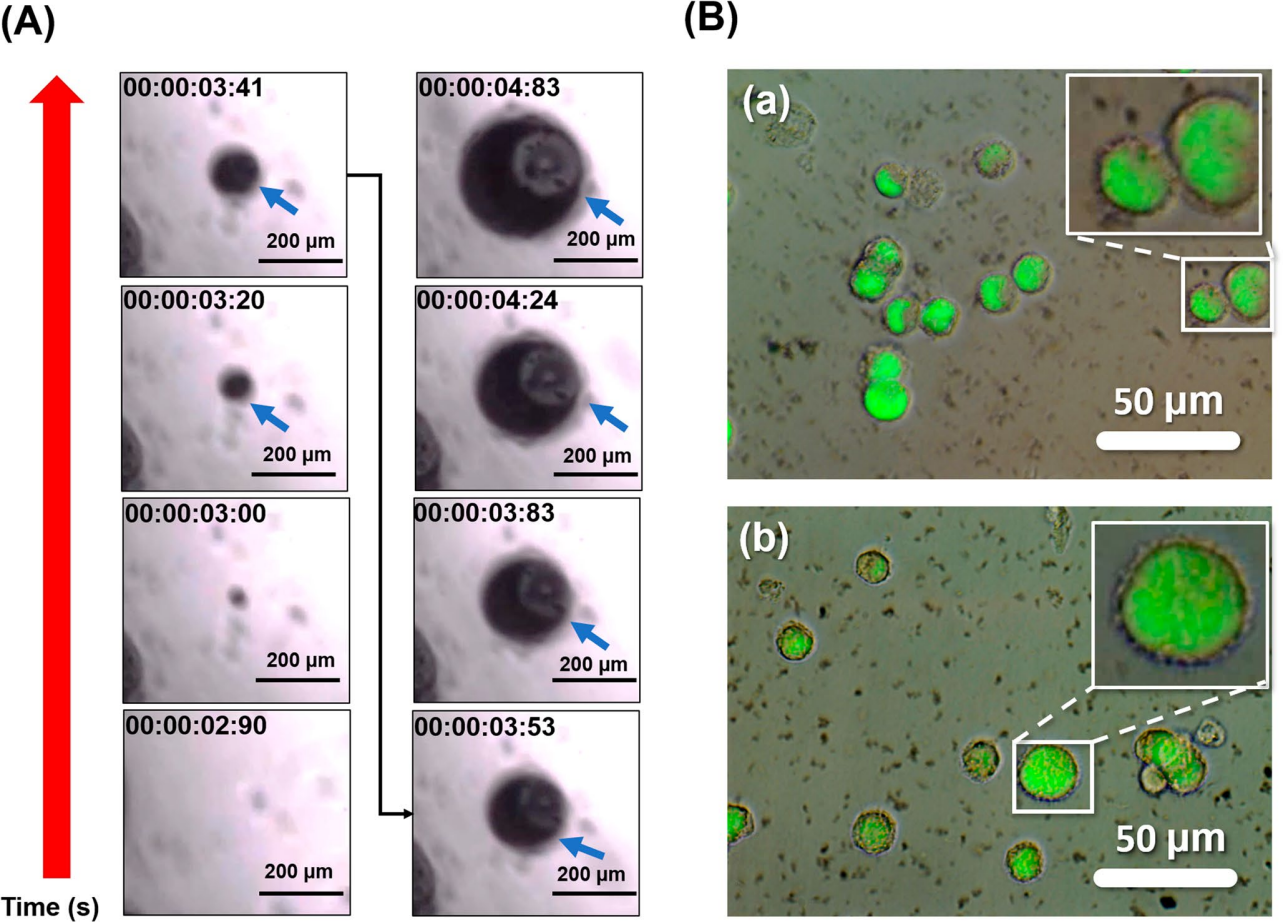
**A** The growing of an O_2_ bubble formed by CAT-like activity of Hb@Ru NPs with the increasing time and the convective motion of Hb@Ru NPs on its surface in the radial direction in the presence of 500 mM H_2_O_2_. Concentration of Hb@Ru NPs: 0.50 mg mL^-1^, 22 °C. **B** The effect of nanomotor function on the interaction of Hb@Ru NPs with T98G cells. The inverted fluorescence microscopy images of (**a**) non-adherent T98G cell suspension containing Hb@Ru NPs, (**b**) non-adherent T98G cell suspension containing Hb@Ru NPs and H_2_O_2_. Concentration of Hb@Ru NPs: 0.5 mg mL^-1^, H_2_O_2_ concentration: 1 mM, T98G cell density: 2 × 10^4^ cells/well, 37 °C, Treatment time: 10 min

The strong CAT-like activity provided a self-propelled motion ability to Hb@Ru NPs. The O_2_ bubbles formed by the decomposition of exogeneous H_2_O_2_ generated a propulsion effect for the self-propelled motion of the Hb@Ru NPs. To explain the nanomotor ability of Hb@Ru NPs, O_2_ bubble generation in the aqueous solution containing Hb@Ru NPs and H_2_O_2_ was monitored under optical microscopy and given in Movie S1. The growing of an O_2_ bubble generated by the CAT-like activity of Hb@Ru NPs with the increasing time and the convective motion of Hb@Ru NPs on its surface in the radial direction in the presence of 5 mM H_2_O_2_ is shown in Fig. 4A. The frames at different times were taken from a video recorded during the growing of a single O_2_ bubble. The interfacial region on the surface of the growing O₂ bubble, appearing as a light grey circle on the bubble surface, should contain Hb@Ru NPs participating into O_2_ evolution reaction via decomposition of H_2_O_2_. Individual Hb@Ru NPs in this region should move in the radial direction at a linear velocity which is equal to the radial expansion rate of growing O_2_ bubble. A growing O_2_ bubble also transfers its momentum to the aqueous dispersion and generates a convective motion in the bulk liquid containing Hb@Ru NPs.

In order to follow H_2_O_2_ fueled-convective motion under an inverted optical microscope, Hb@Ru NPs were dispersed in 1x PBS buffer at pH 7.4 containing H_2_O_2_ in the form of micro-agglomerates only by vortexing the aqueous dispersion without applying ultrasonication. The trajectories of selected micro-agglomerates were marked by a blue tracking line on Movie S2, using Fiji/ImageJ^®^ software. These trajectories showed that the motion of Hb@Ru micro-agglomerates was distinct from Brownian motion. The motion trajectories on XY plane and the linear velocities of selected Hb@Ru micro-agglomerates are given in Figure S8A and Figure S8B, respectively. The linear velocities varying between 65 and 85 μm/s should be explained by self-propelled motion of the Hb@Ru NPs.

To test the effect of self-propelled motion. the interaction between non-adherent T98G cells and Hb@Ru NPs was investigated in the absence and presence of exogeneous H_2_O_2_ in the cell culturing medium, under fluorescence microscope (Fig. 4B). Here, Hb@Ru NPs attached to the cells dyed with AO are observed in the form of yellow micro-agglomerates. By comparing both images, one can see that, higher number of Hb@Ru NPs attached onto the cells in the presence of H_2_O_2_. In order to quantify the effect of H_2_O_2_ on the extent of Hb@Ru NPs attached to and/or uptaken by T98G cells, the cells were interacted with Hb@Ru NPs in the absence and presence of H_2_O_2_ in DMEM. The extent of attached- and/or uptaken- Hb@Ru NPs was determined for both cases via a spectrophotometric protocol given in Section S7. The absorbance obtained with uptaken/attached Hb@Ru NPs with 1 mM H_2_O_2_ in the culturing medium is 1.7 times higher than that obtained in the absence of H_2_O_2_ (Figure S9). These runs supported the hypothesis on the positive contribution of the nanomotor function to the interaction between of Hb@Ru NPs and cells.

The ultrasmall size (~ 10 nm) and protein-functionalized surface of Hb@Ru NPs may favor internalization through classical energy-dependent endocytic pathways, particularly clathrin- or caveolae-mediated mechanisms [26]. Previous studies have demonstrated that nanoparticle size and surface composition are critical determinants of cellular uptake efficiency and intracellular trafficking [27]. In addition, the transient increase in cellular volume observed following treatment may be associated with oxidative stress-induced osmotic dysregulation caused by intracellular nanoparticle accumulation and ROS generation, ultimately contributing to apoptotic cell death [28].

### ROS generation behavior of Hb@Ru NPs

**Fig. 5:**
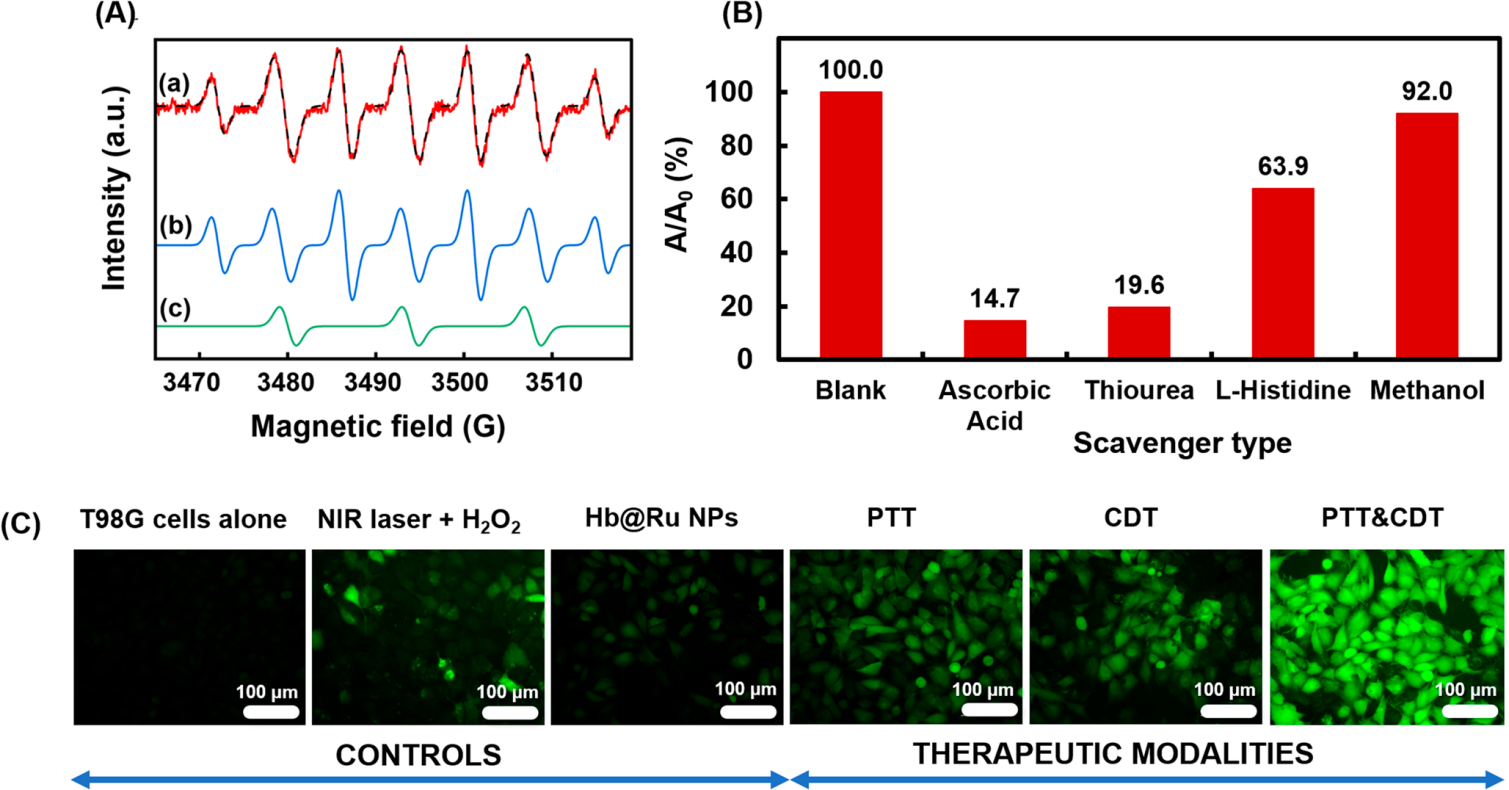
**A** ESR spectra recorded in acetate buffer (200 mM) at pH 5.0 after a reaction period of 5 min, Conditions: Concentration of Hb@Ru NPs: 0.5 mg mL^-1^, H_2_O_2_: 15 mM, Reaction period: 5 min, Spin trap (DMPO): 67 mM, Standard g-marker: DPPH, (**a**) Experimental spectra & simulated curve, predicted curves for (**b**) O_2_^●-^ and (**c**) ^1^O_2_ using fmminisearch^®^ software. **B** Scavenging runs for ROS generated by Hb@Ru NPs. Ascorbic acid (100 mM), thiourea (100 mM), L-histidine (100 mM) and methanol (1.0 mM) were used as the scavengers for O_2_^●-^, ^1^O_2_ and ●OH radicals, respectively. Nanozyme concentration: 1.0 mg mL^-1^. Temperature: 23 °C, Reaction period: 1 min. **C** Intracellular ROS formation by the interaction of Hb@Ru NPs with T98G cells. **a** The control image taken with only cells and with cells in the presence of H_2_O_2_ (1 mM) by applying NIR laser at 808 nm for 5 min, Power density: 1.0 W/cm^2^. **b** The images obtained using Hb@Ru NPs at different therapeutic modes: only Hb@Ru NPs, PTT was performed with NIR laser irradiation at 808 nm in the presence of Hb@Ru NPs (0.5 mg mL^-1^) with T98G cells for 5 min in the absence of H_2_O_2_. CDT was performed in the presence of H_2_O_2_ (1 mM) with Hb@Ru NPs (0.5 mg mL^-1^) and T98G cells for 5 min without applying NIR laser irradiation. PTT&CDT was performed with NIR laser irradiation at 808 nm in the presence of H_2_O_2_ (1 mM) and Hb@Ru NPs (0.5 mg mL^-1^) with T98G cells for 5 min. T98G cell density in all cases: 2 × 10^4^ cells/well

The ESR spectrum of Hb@Ru NPs consists of seven resonance signals with slightly different intensities (Fig. 5A). The signal intensities of the first and seventh signals are slightly weaker than those of other signals. Similar ESR spectra were also observed and originated from the radicals DMPO-O_2_^-●^ and DMPO-(^1^O_2_) [29, 30]. The ESR spectra from the simulation calculations is given in Fig. 5A(a) as dashed lines. ESR spectra generated using the parameters calculated from simulation for O_2_^-●^ and ^1^O_2_ radicals clearly confirms the production of these radicals by Hb@Ru NPs (Fig. 5A (b) and 5A (c)).

The radical scavenging runs performed with Hb@Ru NPs are given in Fig. 5B. The absence of inhibition with methanol indicates that significant ●OH production does not occur. The most significant inhibition observed with both ascorbic acid and thiourea shows that O_2_^●-^ is effectively generated by Hb@Ru NPs. A moderate inhibition with L-histidine suggests that ^1^O_2_ is generated to a less extent with respect to O_2_^●-^. Consequently, the generation of O_2_^●-^ and ^1^O_2_ by Hb@Ru NPs are consistently shown by ESR spectroscopy and radical scavenging runs. The generation of •OH radicals by plain Hb, Plain RuO_2_ and Hb@Ru NPs were also checked using 2-hydroxyterephthalic acid (2-HTPA) florescence assay [23, 29]. No significant •OH radical formation was observed with plain RuO_2_ and Hb@Ru NPs (i.e. Ru based NPs) while a weak •OH generation was observed with plain Hb NPs (Figure S10). Hence, the colorimetric conversion of OPDA via POD-like activity of Hb@Ru NPs can be explained by the role of ^1^O_2_ and O_2_^-•^ radicals in the oxidation reaction [31].

The intracellular ROS generation in the presence of Hb@Ru NPs was studied using DCFDA as the fluorescence probe [23, 29]. No significant fluorescence emission depending on ROS production is observed in the images obtained with the control samples including T98G cells and T98G cells with 1 mM H_2_O_2_ under NIR radiation at 808 nm (Fig. 5C). The images in Fig. 5C obtained with different therapeutic modalities and also control samples were processed by Image-J^®^ software, to quantify the ROS generated (Figure S11). The relative fluorescence intensity obeyed the order of PTT&CDT > PTT>CDT and demonstrated the effectiveness of combinatorial modality (Figure S11).

### Photothermal conversion with Hb@Ru NPs

**Fig. 6:**
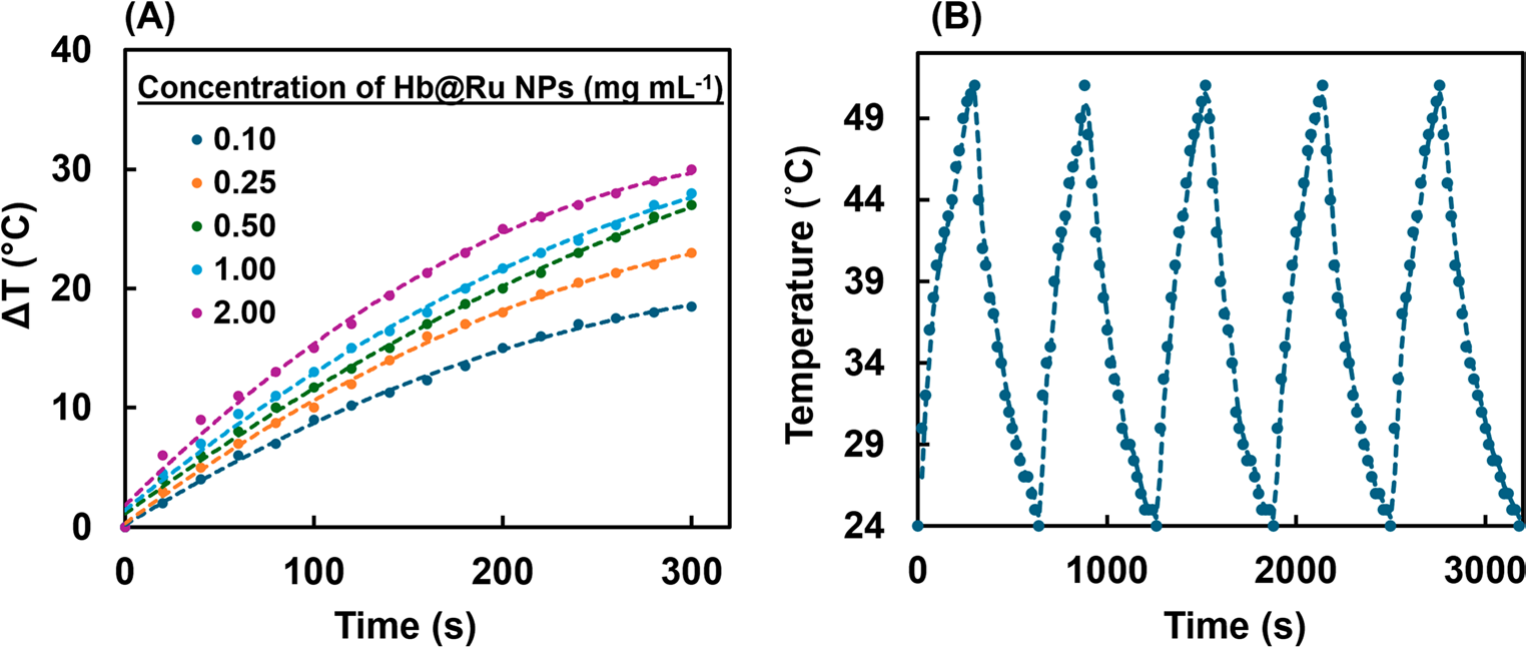
**A** Temperature elevation curves obtained with different concentrations of Hb@Ru NPs. NIR Laser at 808 nm, Power density: 1.0 W/cm^2^, DI water, 200 µL, (**B**) Successive heating-cooling curves by turning on/off of NIR laser in the presence of Hb@Ru NPs. Concentration of Hb@Ru NPs: 1.0 mg mL^-1^

Hb@Ru NPs has photothermal conversion ability under NIR laser irradiation at 808 nm. The temperature elevations recorded with different concentrations of Hb@Ru NPs are given in Fig. 6A. The temperature elevations between 14 and 29 °C were obtained in 300 s by varying the concentration of Hb@Ru NPs between 0.05 and 2.0 mg mL^-1^, under NIR laser irradiation. The successive heating-cooling curves obtained by turning on/off NIR laser placed top of the aqueous dispersion of Hb@Ru NPs are included in Fig. 6B. The consecutive curves in Fig. 6B demonstrated the reversibility of photothermal behavior. The photothermal conversion efficiency and the time constant were calculated as 46.7% and 128.1 s, respectively, by the evaluation of successive heating-cooling curve given in Figure S12A and Figure S12B [32].

The band-gap energy of Hb@Ru NPs was calculated as 1.6 eV by the extrapolation of the linear part of Tauc Plot in Figure S13 [33]. If the photon energy from the laser matches the band-gap energy of the nanomaterial, a strong photon absorption occurs providing a more efficient photothermal conversion leading to an efficient heat generation under irradiation. The photon energy of an 808 nm NIR laser calculated by the Planck–Einstein relation is ~ 1.53 eV [34]. Hence, the band-gap energy of Hb@Ru NPs matches well with the photon energy of an 808 nm NIR laser which explains the apparent photothermal response of Hb@Ru NPs given in Fig. 6. Besides, the absorption spectrum of Hb@Ru NPs was recorded to demonstrate the appreciable light absorption in NIR-I region (700–1000 nm) in which NIR laser at 808 nm was operated (Figure S14).

### In-vitro antitumor activity of Hb@Ru NPs

In the cytotoxicity runs performed with Hb@Ru NPs, the viabilities ranging between 96.6% and 93.1% were found by varying the concentration of Hb@Ru NPs in the range of 0.0–2.0 mg mL^− 1^ with L929 mouse fibroblast cells selected as a healthy cell line (Figure S15). In the cytotoxicity runs made with T98G glioblastoma cells, the viabilities ranging between 99.0% and 92.5% were obtained when the concentration of Hb@Ru NPs was changed in the same range (Figure S16). Hence, no significant cytotoxicity was observed for Hb@Ru NPs for healthy cells and tumor cells. To observe a dose-response behaviour in a broad concentration range extended to the upper threshold of cytocompatibility, the concentration range of 0.0–2.0 mg mL^− 1^ was selected in all therapeutic modalities. Note that exogenous H_2_O_2_ concentration did not cause a remarkable cell death in the T98G culturing medium. The maximum cell death observed with the exogeneous H_2_O_2_ concentration used in these runs (i.e. 1 mM) was ca. 5.0% [23, 29].

**Fig. 7:**
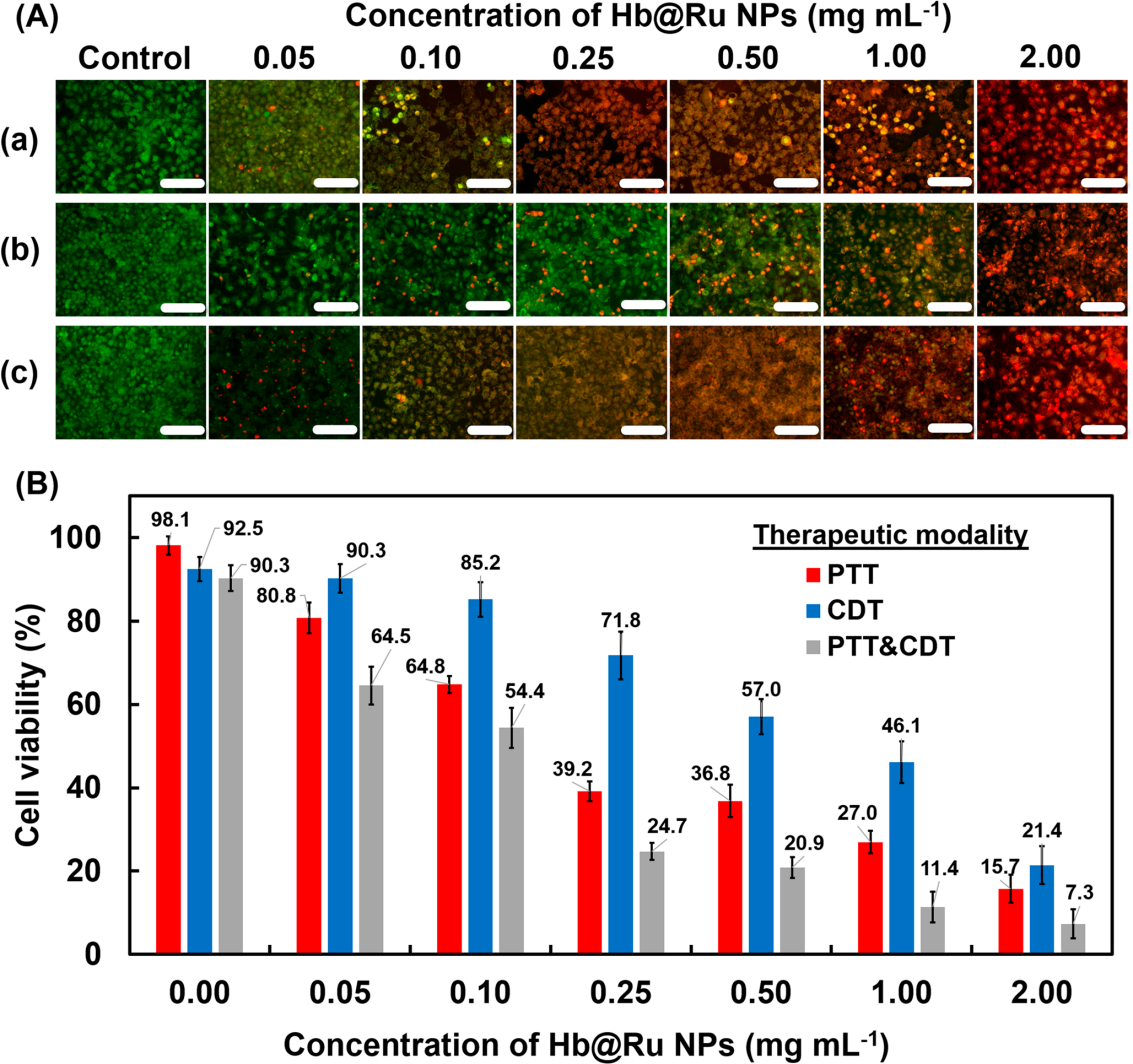
Representative live/dead cell images of T98G cells stained with AO/PI after treatment with different concentrations of Hb@Ru NPs. Panel (**A**): (**a**) PTT modality with different concentrations of Hb@Ru NPs, PTT was applied using NIR laser at 808 nm with a power density of 1 W/cm^2^ in the absence of H_2_O_2_ for 5 min. T98G cell density: 2 × 10^4^ cells/well. (**b)** CDT modality with different concentrations of Hb@Ru NPs, CDT was applied by adding exogeneous H_2_O_2_ at a concentration of 1 mM for 5 min without applying NIR laser. T98G cell density: 2 × 10^4^ cells/well. (**c)** PTT&CDT modality with different concentrations of Hb@Ru NPs, PTT&CDT combination was applied using NIR laser at 808 nm with a power density of 1 W/cm^2^ in the presence of 1 mM exogeneous H_2_O_2_ for 5 min. Scale bar: 200 μm. Panel (**B**): MTT results demonstrating the viability of T98G cells after interaction with Hb@Ru NPs at different concentrations in different therapeutic modalities. Number of replicates: 3. Mean ± SD

Representative live/dead cell images of T98G cells stained with AO/PI after interaction with Hb@Ru NPs at different concentrations using different therapeutic modalities including PTT, CDT and PTT&CDT are presented in Fig. 7A. MTT results showing the viability of T98G cells in these interactions are given in Fig. 7B. As seen in both Fig. 7A and B, appreciable cell deaths are observed with Hb@Ru NP concentrations higher than 0.1 mg mL^-1^ in PTT mode alone. However, higher concentrations of Hb@Ru NPs are needed to achieve significantly high cell deaths in CDT mode alone. Intracellular ROS production with an exogeneous H_2_O_2_ concentration of 1 mM in the cell culturing medium was more effective on the cell death with Hb@Ru NP concentrations higher than 0.5 mg mL^-1^. Combined PTT&CDT modality was applied by exposing the cell culturing medium containing exogeneous H_2_O_2_ to NIR laser irradiation. Here, considerable cell deaths were obtained with Hb@Ru NP concentrations higher than 0.25 mg mL^-1^. The increased ROS generation at higher temperatures, induced by photothermal conversion capability of Hb@Ru NPs should explain the higher cell deaths achieved with combined PTT&CDT treatment compared to PTT alone or CDT alone.

**Fig. 8:**
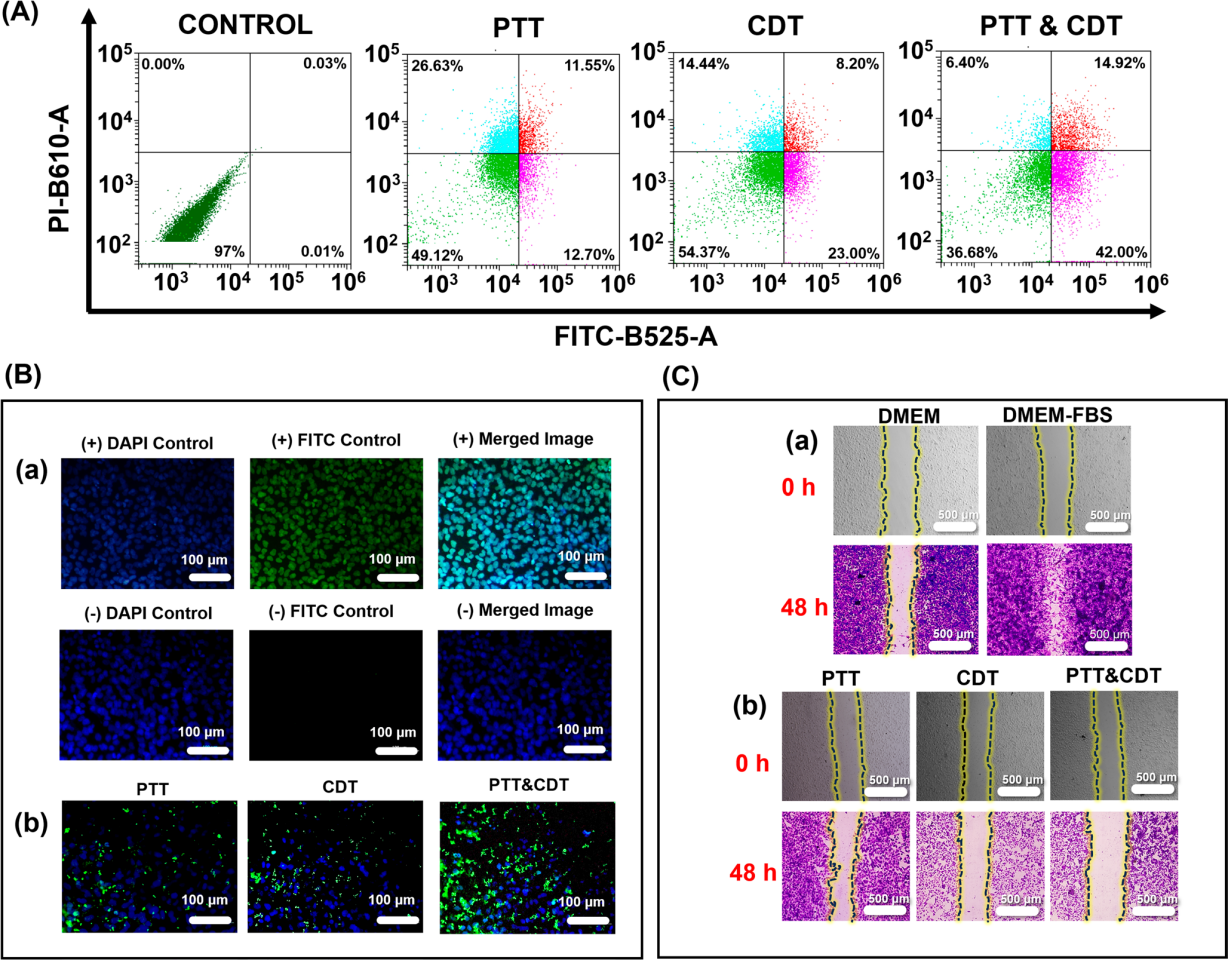
Panel (**A**): Flow cytometry analysis of T98G cells following interaction with Hb@Ru NPs with different therapeutic modalities given as control sample, PTT, CDT, and PTT&CDT, respectively. The cells were stained with Annexin V-FITC and PI to observe viable cells (Annexin V−/PI−), early apoptotic cells (Annexin V+/PI−), late apoptotic cells (Annexin V+/PI+), and necrotic cells (Annexin V−/PI+) in the quadrants. Concentration of Hb@Ru NPs: 0.5 mg mL^-1^. Panel (**B**): The images of apoptotic T98G cells exposed to TUNEL assay. Conditions: T98G concentration: 2 × 10^4^ cells/well, Concentration of Hb@Ru NPs: 0.5 mg mL^-1^. The therapeutic combinations are included with the images. **a**: Control groups for T98G cells: (+) DAPI positive control, (+) FITC positive control, (+) Merged image and (-) DAPI negative control, (-) FITC negative control, (-) Merged image. **b** Different therapeutic modalities with Hb@Ru NPs. The therapeutic modality applied under the conditions given in the legend of Fig. 7 is given on the image. Panel (**C**): The inverted microscopy images of T98G cells for monitoring proliferation and migration of T98G cells in the SCRATCH assay (**a**): Control groups including DMEM without FBS, DMEM with FBS, (**b**) The inverted microscopy images of T98G cells taken initially and at 48 h with the interaction with Hb@Ru NPs via different therapeutic modalities applied under the conditions given in the legend of Fig. 7 is given on the image. The concentration of Hb@Ru NPs: 0.5 mg mL^-1^, T98G concentration: 2 × 10^5^ cells/well

In PTT&CDT combination, the maximum cell death for T98G cells was obtained as 92.7% with the Hb@Ru NP concentration of 2.0 mg mL^-1^. Remarkable cell deaths with PTT&CDT modality with respect to those of PTT alone or CDT alone demonstrate the synergistic character of this combination. The flow cytometry analysis of T98G cells after interaction with Hb@Ru NPs with different therapeutic modalities is given in Fig. 8A. The PTT&CDT combination with Hb@Ru NPs resulted in a substantially higher apoptotic rate (63.32%) compared with CDT alone (45.63%) or PTT alone (50.88%). These results indicate a pronounced synergistic effect of PTT&CDT with Hb@Ru NPs.

Hb@Ru NPs was also tried using HepG2 cells as a widely used human liver cancer cell line. The live/dead HepG2 cell images and MTT results given in Figure S17 revealed that the cell viability was reduced up to 6.0% with the increasing concentration of Hb@Ru NPs in the range of 0.05-2.0 mg mL^-1^. These results obtained with another tumor cell line confirmed the effectiveness of Hb@Ru NPs in the combinatorial, in-vitro PTT&CDT application.

TUNEL staining was performed to evaluate DNA fragmentation as an indicator of apoptosis in T98G cells interacted with 0.5 mg mL^-1^ Hb@Ru NPs under different therapeutic modalities. In the negative control group, nuclei were uniformly stained with DAPI (blue) with negligible FITC signal, confirming the absence of spontaneous DNA fragmentation and low background staining (Fig. 8B). The positive control group with artificially induced DNA breaks exhibited intense FITC fluorescence (green), validating the efficiency and specificity of TUNEL assay (Fig. 8B), The merged images demonstrated clear colocalization of DAPI and FITC signals in TUNEL-positive cells. Following treatment with Hb@Ru NPs (0.5 mg mL^-1^), apoptotic cells were identified by green nuclear fluorescence. PTT and CDT groups showed an increase in FITC-positive cells compared to the negative control, indicating induction of apoptosis. Notably, the PTT&CDT combination exhibited the highest density and distribution of TUNEL-positive cells (Fig. 8B). Hence, Hb@Ru NPs effectively induces DNA fragmentation in T98G cells, with PTT&CDT treatment producing the most pronounced apoptotic response.

The synergistic effect of Hb@Ru NPs on glioblastoma cell migration and proliferation was evaluated using a wound healing (scratch) assay (Fig. 8C). The wound closure process, driven by cell migration and proliferation, was monitored for 48 h under different therapeutic modalities using an inverted phase-contrast optical microscope. The wound closure plot constructed based on the inverted optical microscopy images in Fig. 8C is given in Figure S18. Here, the wound closure values at 48 h were determined by the comparative usage of Image J^®^ wound healing size tool for the images taken initially and at 48 h. The applied treatments included PTT, CDT, and CDT/PTT. At the end of the 48-hour incubation period, the scratched regions were stained with Crystal Violet to further evaluate cell density and wound closure. No significant wound closure was observed in the FBS-free control group within 48 h (Fig. 8C and Figure S18). In contrast, pronounced wound closure was detected in the FBS-containing control group, reflecting the intrinsic proliferative and migratory capacity of glioblastoma cells (Fig. 8C and Figure S18). The application of Hb@Ru NPs in combination with PTT resulted in a noticeable delay in the wound closure process compared with the control groups (Fig. 8C and Figure S18). This finding suggests that the application of Hb@Ru NPs enhanced the photothermal conversion efficiency, thereby effectively suppressing the migration and proliferation of glioblastoma cells. When Hb@Ru NPs was applied together with CDT or CDT/PTT modality, the inhibitory effect became much more pronounced. The wound healing process was largely suppressed after 48 h, and the scratched areas remained substantially open. These results were further confirmed by staining with Crystal Violet, which revealed a marked reduction in staining intensity and an increase in cell-free regions. Microscopic observations showed a reduction in cell–cell interactions and disruptions in cellular morphology in certain regions. These morphological alterations indicate that Hb@Ru NPs effectively suppress the migration and proliferation capacity of glioblastoma cells under synergistic therapeutic conditions, thereby significantly inhibiting the wound closure process.

Flow cytometric analysis of cell cycle distribution in T98G cells given in Figure S19 showed that the Sub-G1 region, located to the left of the G0/G1 peak, increased following treatment in different modalities, indicating apoptotic DNA fragmentation and cell death. Control cells displayed a predominantly proliferative profile with a high S-phase population. In contrast, both PTT and CDT treatments caused significant G2/M accumulation accompanied by reduced S-phase proportions, consistent with activation of DNA damage-associated cell cycle arrest and suppression of proliferation. Notably, the combined PTT&CDT treatment produced the highest Sub-G1 population, suggesting enhanced apoptotic cell death under severe oxidative and thermal stress. These findings demonstrate the strong antiproliferative and cytotoxic effects of the combined therapy. Consequently, only PTT and only CDT induced G2/M accumulation, suggesting DNA damage-associated cell cycle arrest. In contrast, combined CDT&PTT markedly increased the Sub-G1 population while reducing G2/M proportions, indicating enhanced apoptotic cell death under severe oxidative and thermal stress conditions.

## Conclusion

A new and single-stage hydrothermal protocol was proposed for synthesis of Hb@Ru NPs with a substantial Ru content in the crystalline form. In principle, the developed method can be applied for the synthesis of Hb based NPs containing different noble metals such as Ag, Pt, Au, Pd, Rh and Ir that may potentially act nanozymes with strong enzyme-mimetic activities. In the proposed method, Ru was selected as an appropriate metal atom to provide a superior enzyme-mimetic activity and strong ROS production ability. The presence of Ru atoms endowed the nanostructure with strong CAT-like activity, which underlies its nanomotor function. Hence, Hb@Ru NPs is designed as a self-propelled nanozyme capable of autonomous motion in tumor microenvironment to achieve more effective interaction with the tumor cells. The strong CAT-like activity of self-propelling nanozyme allows the alleviation of hypoxia and the application of more effective CDT against tumor cells. Photothermal conversion ability of Hb@Ru NPs is another superiority allowing to apply a more effective CDT. An increase should occur in the generation rate of ROS by the temperature elevation in the tumor microenvironment which allows the amplification of DNA damage in tumor cells as demonstrated by TUNEL assay in PTT&CDT application with Hb@Ru NPs. The photothermal conversion ability should also enhance GSH depletion ability of Hb@Ru NPs. The usage of HB@Ru NPs in the combinatorial PTT&CDT application with glioblastoma cells resulted in-vitro cell deaths higher than 90%. Hence, Hb@Ru NPs can be termed as a new and promising nanoplatform for the synthesis of novel therapeutic agents for either antitumor or antibacterial applications. The data collected with in-vitro studies in this work will build a reliable framework for the systematic design of subsequent in vivo studies.

## Supplementary Information

Supplementary Material 1.

## Data Availability

The data that support the findings of this study will be made available on request.
