# Supplementary material for: A hemoglobin-based nanozyme with ruthenium-induced nanomotor ability exhibiting photothermal and chemodynamic responses
Source: Mikrochim Acta. 2026 Jun 10;193(7):463. doi: 10.1007/s00604-026-08176-3 (PMC13253789; doi:10.1007/s00604-026-08176-3)
Supplement: Supplementary file 1 — Supplementary Material 1. [file 604_2026_8176_MOESM1_ESM.docx]

**A hemoglobin-based nanozyme with ruthenium-induced nanomotor ability exhibiting photothermal and chemodynamic responses**

*Gökçe Çoban^1,2^, Esin Akbay Çetin^3^, İrem Yağmur Gök^3^, Neşen Betül Kaya^1^,*

*Burcu Gökçal Kapucu^1^, Mehmet Ali Onur^3,4^, Mustafa Polat^5^,*

*Çiğdem Kip^1,^*, Ali Tuncel^1,4,^**

^1^ Hacettepe University, Chemical Engineering Department, Ankara 06800, Turkey

^2^ Hacettepe University, Graduate School of Science & Engineering, Ankara 06800, Turkey.

^3^ Hacettepe University, Department of Biology, Ankara, 06800, Turkey.

^4^ Hacettepe University, Division of Bioengineering, Graduate School of Science & Engineering, Ankara 06800, Turkey.

^5^ Hacettepe University, Department of Physics Engineering, Ankara, 06800, Turkey.

***Corresponding author**: Ali Tuncel, Ph.D. [atuncel@hacettepe.edu.tr](mailto:atuncel@hacettepe.edu.tr) and Çiğdem Kip, Ph.D. [cigdemg@hacettepe.edu.tr](mailto:cigdemg@hacettepe.edu.tr)

**Supporting Information**

**S1. EXPERIMENTAL**

**S1.1. Materials**

Hemoglobin from bovine blood (lyophilized powder), ruthenium (III) chloride hydrate, sodium hydroxide, 3,3′,5,5′-tetramethylbenzidine (TMB), o-phenylenediamine (OPDA), hydrochloric acid (HCl, 37.5% w w^-1^), titanium (IV) chloride (TiCl_4_), hydrogen peroxide (H_2_O_2_, 50% w w^-1^), ethanol, tris(hydroxymethyl)aminomethane, terephthalic acid (TPA), L-glutathione reduced, 2',7'-dichlorodihydrofluorescein diacetate (DCFDA), 5,5′-dithiobis(2-nitrobenzoic acid) (DTNB), acetic acid, 5,5-dimethyl-1-pyrroline-N-oxide (DMPO), sodium acetate trihydrate, dimethyl sulfoxide (DMSO), L-histidine, thiourea, ascorbic acid and terephthalic acid were purchased from Sigma-Aldrich (St. Louis, MO, USA). Di-sodium hydrogen phosphate and methanol were obtained from Merck, Darmstadt, Germany. Sodium phosphate monobasic was obtained from Riedel-de Haën, Seelze, Germany. DMEM/F-12 and fetal bovine serum (FBS) were supplied by Biochrom AG, Berlin, Germany. Penicillin/streptomycin (P/S), 0.25% trypsin–EDTA, propidium iodide (PI), phosphate buffered saline (PBS), acridine orange (AO) and 3-(4,5-dimethylthiazol-2-yl)-2,5-diphenyl tetrazolium bromide (MTT) were used without further purification. L929 (Subcutaneous connective tissue cells), T98G (human glioblastoma cells) and Hepatoma G2 (HepG2, Human hepatocellular carcinoma cells) cell lines were supplied by American Type Culture Collection (ATCC, VA, USA). Deionized water (DI) (18 MΩ cm) was produced using a Direct-Q3, Millipore, U.S.A.

**S1.2. Preparation of Hb@Ru NPs**

Hb@Ru NPs was synthesized using a single-stage hydrothermal protocol. Typically, Hb (50 mg) and the metal salt (RuCl_3_.hydrate, 170 mg) were dissolved in DI water (20 mL). The medium was magnetically stirred at ambient temperature for 2 h, then placed in a Teflon-lined autoclave and maintained at 140°C for 12 h. The reactor was cooled down to room temperature. Hb@Ru NPs was washed three times with DI water. The sample was dried overnight at 60°C and stored for further use. Plain Hb NPs was synthesized as a control via hydrothermal treatment of Hb in the absence of Ru salt under the same conditions. Similarly, plain (hydrous) RuO_2_ NPs was synthesized as the another control via hydrothermal treatment of RuCl_3_.H_2_O in the absence of Hb under the same conditions.

**S1.3. Characterization of Hb@Ru NPs.**

The morphology and the size characteristics of Hb@Ru NPs were analyzed using transmission electron microscopy (TEM, JEOL, JEM-2100, U.S.A.). The crystalline phase and structure of nanoparticles (NPs) were characterized by X-ray diffraction spectroscopy (XRD, Rigaku Ultima-IV, Japan) in the 2θ range of 10–100° with a scanning rate of 2° min^-1^. The hydrodynamic size distribution of Hb@Ru NPs was determined by Dynamic Light Scattering (DLS) (Malvern Instruments Zetasizer Nano-S, U.K.). To determine their zeta potentials, Hb@Ru NPs (5 mg L^-1^) was dispersed in DMEM in the pH range of 5-8. X-ray Photoelectron Spectroscopy (XPS, K-Alpha system, Thermo Fischer Scientific, U.S.A.) was used to analyze the surface chemistry of Hb@Ru NPs. The metal content of Hb@Ru NPs was determined using inductively coupled plasma–optical emission spectroscopy (ICP-OES instrument, Varian/Agillent, 720 ES, U.S.A). Thermogravimetic analysis (TGA) of Hb@Ru NPs was performed in TG/DTA-Seiko Instruments SII, Exstar 6300 (Tokyo/Japan).

**S1.4. ESR Spectroscopy and Radical Scavenging Runs**

The generation of O_2_^●-^ and ^1^O_2_ radicals by Hb@Ru NPs was determined using electron spin resonance (ESR) spectroscopy with DMPO as the spin trap and 1,1-diphenyl-2-picrylhydrazyl (DPPH) as the standard g-marker. For the detection of O_2_^●-^ and ^1^O_2_ radicals, Hb@Ru NPs (1 mg) was added to a methanol solution (2 mL) containing H_2_O_2_ (1 μL). After 5 min of reaction, the mixture was centrifuged and DMPO (100 mM) was added to the supernatant. ESR spectra was obtained at room temperature using a Bruker EMX-131 X-band ESR spectrometer equipped with a standard cylindrical cavity (Bruker ER 4102ST). The central magnetic field, microwave frequency and microwave power were set to 3485 G, 9.8 GHz, 16 mW, respectively. The modulation amplitude, the modulation frequency, the scan amplitude was 2.0 G, 100 kHz, 150 G, respectively. The time constant and the scan time were 81.92 ms, 83.89 s, respectively. The same protocol was also applied for the detection of •OH radicals, using acetate buffer (pH 5.0) instead of methanol as the reaction medium.

The simulation calculation was performed with the non-linear least-squares solver in MATLAB to calculate the ESR parameters of produced radicals. From the simulation calculations performed the hyperfine splitting constant of A_N_= 13.9 G for DMPO-(^1^O_2_) and A_N_= 14.6 G, AHα= 7.7 G and AHβ= 6.7 G for DMPO-O_2_^-•^ were determined. The Landé-g factor was found to be 2.0048 for both radicals detected.

In the scavenging runs, the presence of three distinct radicals (O_2_^●-^, •OH and ^1^O_2_) was investigated. Ascorbic acid, methanol, and L-histidine were utilized to capture O_2_^●-^, •OH and ^1^O_2_ radicals, respectively. To determine O_2_^●-^ radical, ascorbic acid (100 mM) was dissolved in a Tris-buffer solution (10 mM, pH 7.0) containing o-phenylenediamine (OPDA, 7.5 mM), H_2_O_2_ (17.5 mM), and Hb@Ru NPs (1 mg mL^-1^). The same protocol was also applied by dissolving L-histidine (100 mM) instead of ascorbic acid in Tris-buffer for the detection of 1O2 radical. At the end of the prescribed reaction period (1 min), the aqueous dispersion was subjected to centrifugation at 10000 rpm for 15 min. The presence of coloured product, 2,3-diaminophenazine in the supernatant was analyzed by measuring the absorbance against time at 416 nm in a UV–Vis spectrophotometer (Thermo Scientific, Genesys 150, USA) [S1, S2].

A similar protocol was also followed for the determination of •OH radical using MeOH (100 mM). In this case, the formation of oxidized product was followed in an acetate buffer solution at pH 5.0 containing Hb@Ru NPs (1 mg mL^-1^), H_2_O_2_ (17.5 mM) and TMB (0.5 mM). At the end of the prescribed reaction period (1 min), the aqueous dispersion was centrifuged at 10000 rpm for 15 min. The presence of coloured product, oxidized-TMB in the supernatant was analyzed by measuring the absorbance against time at 652 nm in a UV–Vis spectrophotometer [S1, S3].

**S1.5. Peroxidase-like (POD-like) Activity of Hb@Ru NPs**

O-phenylenediamine (OPDA) was selected as the chromogenic substrate to evaluate the peroxidase-like (POD-like) activity of Hb@Ru NPs. For POD-like activity, the kinetics of OPDA oxidation in the presence of H_2_O_2_ was investigated using the Michaelis–Menten model. The reaction was conducted in a Tris buffer solution (50 mM, pH 7.0) at ambient temperature in a reaction volume of 2.0 mL. The initial OPDA concentration was varied from 100 to 10000 μM in the presence of Hb@Ru NPs (1.0 mg mL^-1^). To determine the OPDA consumption rate for each OPDA initial concentration, the absorbance of the supernatant obtained by the centrifugation of an aqueous sample withdrawn from the dispersion was measured at 412 nm. The initial OPDA consumption rate was calculated according to the expression given previously [S1, S3].

**S1.6. Catalase-like (CAT-like) Activity of Hb@Ru NPs**

Catalase-like (CAT-like) activity of Hb@Ru NPs was evaluated by measuring the decomposition of H_2_O_2_. The initial H_2_O_2_ concentration was varied from 0.5 to 150 mM in an aqueous solution containing Hb@Ru NPs (0.05 mg mL^-1^). The reaction was conducted at ambient temperature in a reaction volume of 2.0 mL. After a reaction time of 1 min, the sample (2 mL) was centrifuged at 14000 rpm to remove Hb@Ru NPs. Subsequently, TiCl_4_ solution (600 μL) was added to the supernatant (0.6 mL), and the absorbance of resulting solution was recorded at 414 nm to quantify the residual H_2_O_2_. The initial H_2_O_2_ consumption rate was calculated according to the expression given previously [S1, S3].

**S1.7. GSH Depletion by Hb@Ru NPs**

Hb@Ru NPs was dispersed in GSH solution (0.5 mM) prepared with phosphate buffer (50 mM, pH 7.0, 5 mL). The mixture was kept at 37 °C and stirred in the dark. At different intervals of time (0, 10, 20, 30 and 60 min), the aliquots were collected and centrifuged to separate Hb@Ru NPs. Subsequently, 50 μL of DTNB solution (10 mM) was mixed with diluted supernatant (500 μL). The concentration of remaining GSH was determined by a UV–vis spectrophotometer (Thermo Scientific, USA) by measuring absorbance at 412 nm [S1, S2].

**S1.8. Evaluation of Photothermal Performance**

The photothermal performance of Hb@Ru NPs was evaluated in an aqueous dispersion (200 μL) containing Hb@Ru NPs (1.0 mg mL^-1^) irradiated with an 808 nm near-infrared (NIR) laser at a power density of 1.0 W cm^-2^ in a microwell plate. The irradiation was continued for 5 min and the temperature of the aqueous dispersion was measured against time using a thermocouple (UNI-T, K-Type Thermocouple Probe). To assess the photothermal stability of Hb@Ru NPs, the aqueous dispersion was cooled down to room temperature, and the heating-cooling process was repeated for five consecutive cycles. The photothermal conversion efficiency was estimated using the method described previously [S1, S3-S5].

**S1.9. Evaluation of Nanomotor Function**

The nanomotor activity of Hb@Ru NPs was evaluated using optical microscopy. Hb@Ru NPs was dispersed in an aqueous solution (0.5 mg mL^-1^) and sonicated until a homogeneous dispersion was obtained. Subsequently, the dispersion (50 μL) was transferred to a 96-well plate, and concentrated H_2_O_2_ (2 μL) was introduced. To analyze the effect of O2 bubbles generated by the catalase-like activity on the convective motion of Hb@Ru NPs, the generation and the growth of O_2_ bubbles were monitored against time using an inverted optical microscope (Olympus IX73, Japan) equipped with a 40x objective [S1, S3, S5].

**S1.10. Fluorescence assay for testing the generation of •OH radicals by plain Hb, Plain RuO_2_ and Hb@Ru NPs**

The generation of •OH radicals by plain Hb, plain RuO_2_ and Hb@Ru NPs in the presence of H_2_O_2_ was investigated by means of a fluorescent assay in which 2-hydroxyterephthalic acid (2-HTPA) was used as the probe [S3]. The reaction medium was prepared by dispersing plain Hb, plain RuO_2_ and Hb@Ru NPs in 200 mM acetate buffer at pH 5.0 (12 mL) containing terephthalic acid (0.5 mM) and H_2_O_2_ (5.0 mM). The dispersion was magnetically stirred in the dark for 1 h at 300 rpm, at 37 °C for the formation of a fluorescent product, 2-HTPA via the reaction between TPA and •OH radicals generated. The NPs was separated from the aqueous part by centrifugation. The fluorescence spectrum of supernatant was obtained in a fluorescence spectrophotometer (Shimadzu, RF-5301PC, Japan) with the excitation at a wavelength of 315 nm. The aqueous solution prepared by dissolving 0.5 mM terephthalic acid and 5.0 mM H_2_O_2_ in DI water (12 mL) was used as a reference.

**S1.11. In Vitro Antitumor Activity of Hb@Ru NPs**

Human glioblastoma T98G cells were used to assess the antitumor efficacy of Hb@Ru NPs. Cells were plated in 96-well plates at a high density (2 x 10⁴ cells per well) and maintained in DMEM supplemented with 10% FBS and 1% penicillin–streptomycin at 37°C under a humidified atmosphere containing 5% CO_2_ for 48 h prior to the treatment. Three different therapeutic modalities photothermal therapy (PTT), chemodynamic therapy (CDT), and combined PTT&CDT treatment were investigated. For PTT treatment, T98G cells were incubated with Hb@Ru NPs at different concentrations and subjected to NIR laser irradiation (808 nm) with a power density of 1.0 W cm^-2^ for 5 min. For CDT treatment, the cells were exposed to Hb@Ru NPs at different concentrations in the presence of exogenous H_2_O_2_ (1 mM) for 5 min without NIR laser irradiation. For the combined PTT&CDT treatment, the cells were incubated with Hb@Ru NPs at different concentrations and H_2_O_2_ (1 mM) followed by NIR laser irradiation (1.0 W cm^-2^) for 5 min. After completion of treatment in each modality, the medium was replaced, and the cells were incubated for 48 h. The cell death was qualitatively evaluated using acridine orange/propidium iodide (AO/PI) double staining. Briefly, 100 μL of AO/PI solution (1:1, v v^-1^) was introduced to the cells and the cells were incubated for 2 min, and washed with PBS. The cell images were taken using a fluorescence microscope (Olympus IX70, Japan). The cell viability was also quantitatively assessed using MTT assay. Following treatment, MTT solution (10%, w w^-1^; 200 μL) was introduced to each well, followed by incubation for 4 h. The formazan crystals formed were dissolved in isopropyl alcohol (200 μL), and the absorbance at 570 nm was measured with a microplate reader (μQuant™, BioTek, USA).

To evaluate the biosafety of Hb@Ru NPs, the cytotoxicity studies were conducted using a healthy cell line, L929 mouse fibroblast cells and the selected tumor cells (T98G glioblastoma cells) without applying any therapeutic effect. In these runs, the cells were treated with Hb@Ru NPs by changing the concentration of Hb@Ru NPs between 0.05 and 2.0 mg mL^-1^. The cell viability was assessed by AO/PI staining and MTT assay as described above.

The experimental protocol followed for the determination of In vitro antitumor activity of Hb@Ru NPs was also applied using HepG2 cells via PTT@CDT modality.

**S1.12. Intracellular ROS Generation**

2′,7′-dichlorodihydrofluorescein diacetate (DCFHDA) was employed as the fluorescent probe to evaluate the generation of intracellular reactive oxygen species (ROS). T98G cells were seeded in 96-well plates at a density of 2x10^4^ cells per well and incubated overnight. The cells were treated with Hb@Ru NPs (0.5 mg mL^-1^) using only PTT, only CDT and combined PTT&CDT modalities. After completion of each modality, the cells were incubated with DCFHDA solution (10 μM) at 37 °C for 30 min in the dark. The cells were then washed three times with PBS to remove excess probe. The generation of intracellular ROS was examined using a fluorescence microscope (Olympus IX70, Japan).

**S1.13. Scratch Assay for Cell Proliferation and Migration**

T98G cells were seeded into 24-well plates at a concentration of 2x10^5^ cells well^-1^ and cultured for 24 h. When the confluence level reached 80%, the scratch wounds were created on the monolayer using a 200 μL sterilized pipette tip. Following PBS washing, the cells were treated with Hb@Ru NPs (0.5 mg mL^-1^) in different therapeutic modalities. To evaluate the effect of PTT on cell proliferation and migration, an 808 nm wavelength NIR laser was applied to the cells for 5 min. For the assessment of CDT effect, the medium was exposed to exogenous H_2_O_2_ (1 mM) for 5 min without NIR laser irradiation. For the assessment of combined PTT&CDT effect, both NIR laser irradiation and exogenous H_2_O_2_ exposure were performed under the above conditions. Following the therapeutic application, treated cells and control cells in DMEM medium containing 10% FBS were incubated for 48 h. The cell images were recorded at 0, 6, 12, 24, 36, and 48 h using an inverted microscope to examine cell proliferation and migration. To observe wound closure behavior, the cells were chemically fixed with paraformaldehyde solution (3.7%w w^-1^) after the removal of the medium and permeabilized using methanol. Subsequently, they were stained with crystal violet (1%w w^-1^). After removing excess dye and Hb@Ru NPs with PBS solution, the cell proliferation and migration was imaged using an inverted microscope (Olympus IX70 Inverted Microscope, Japan) [S3].

**S1.14. TUNEL Assay**

T98G cells were seeded in 96-well plates at a density of 2x104 cells well^-1^. Apoptosis was assessed using the terminal deoxynucleotidyl transferase–mediated dUTP nick-end labeling (TUNEL) assay providing an information on DNA fragmentation associated with apoptosis. The cells were treated with Hb@Ru NPs (0.5 mg mL^-1^) under photothermal therapy (PTT), chemodynamic therapy (CDT), and combined PTT&CDT conditions as described above. After treatment, the cells were incubated at 37°C for 48 h and subsequently fixed with a 1% w w^-1^ paraformaldehyde solution. At the end of the incubation period, the ApopTag apoptosis detection kit (Millipore) was utilized to stain the apoptotic cells. The stained cells were imaged using an inverted microscope to evaluate apoptosis levels [S3].

**S1.15. Cell Apoptosis**

For flow cytometry analysis, the T98G cells were seeded in a 6-well plate at 2x105 cells well^-1^  and incubated at 37 °C for 24 h. Then, Hb@Ru NPs (0.5 mg mL^-1^) were interacted with the cells using the therapeutic modalities mentioned above (i.e. only PTT, only CDT and combined PTT&CDT). Then the cells were further incubated at 37 °C for 48 h. Subsequently, the cells were detached from the plate using trypsin and washed with PBS. After centrifugation, Annexin V-FITC (5 μL) and PI (5 μL) were introduced to Annexin V binding buffer (100 μL). Following incubation in the dark for 20 min, the samples were examined by flow cytometry (Beckman Coulter, CytoFLEX LX Flow Cytometer, USA) [S3].

**S.1.16. Flow Cytometric Analysis of Cell Cycle Distribution in T98G Cells**

T98G cells treated with Hb@Ru NPs under different therapeutic conditions were harvested by trypsinization and centrifugation, then resuspended in culture medium at a density of 2 × 10^5^ cells mL^-1^. CytoPhase™ Violet (BioLegend) was added to a final concentration of 10 µM (1:500), and samples were incubated at 37°C for 90 min before analysis using a CytoFLEX LX Flow Cytometer (Beckman Coulter Life Sciences).

**S2. Charaterization of Hb@Ru NPs**

**Table S1.** The structural parameters of plain RuO_2_ and Hb@Ru NPs determined by XRD spectroscopy.

| **Nanoparticle** | **Peak Number** | **2Θ**  **(°)** | **a**  **(nm)** | **b (nm)** | **c**  **(nm)** | **V**  **(nm^3^)** | **d-spacing (nm)** | **Crystallite Size**  **(nm)** | **(hkl)** |
| --- | --- | --- | --- | --- | --- | --- | --- | --- | --- |
| ^a^Plain RuO_2_ | 1 | 28.33 | 0.45 | 0.45 | 0.302 | 0.0598 | 0.3148 | 1.190 | (110) |
|  | 2 | 36.41 | 0.45 | 0.45 | 0.296 | 0.0587 | 0.2466 | 1.200 | (101) |
|  | 3 | 54.89 | 0.45 | 0.45 | 0.307 | 0.0609 | 0.1671 | 1.197 | (211) |
|  | Mean |  | 0.45 | 0.45 | 0.302 | 0.0598 | 0.2428 |  |  |
| Hb@Ru | 1 | 37.88 | 0.27 | 0.27 | 0.442 | 0.0862 | 0.2373 | 9.000 | (100) |
|  | 2 | 43.13 | 0.27 | 0.27 | 0.447 | 0.0872 | 0.2096 | 2.960 | (101) |
|  | 3 | 55.93 | 0.27 | 0.27 | 0.455 | 0.0887 | 0.1643 | 4.200 | (102) |
|  | 4 | 68.35 | 0.27 | 0.27 | 0.442 | 0.0862 | 0.1371 | 5.200 | (110) |
|  | 5 | 84.3 | 0.24 | 0.27 | 0.455 | 0.0887 | 0.1148 | 2.270 | (201) |
|  | Mean |  | 0.274 | 0.27 | 0.448 | 0.0874 | 0.1726 | 4.726 |  |

a: The planes in XRD spectra of Plain RuO_2_ NPs in tetragonal structure were extracted from the literature and the structural constants (a, b, c and crystal volume) were calculated based on tetragonal structure [S6].

**
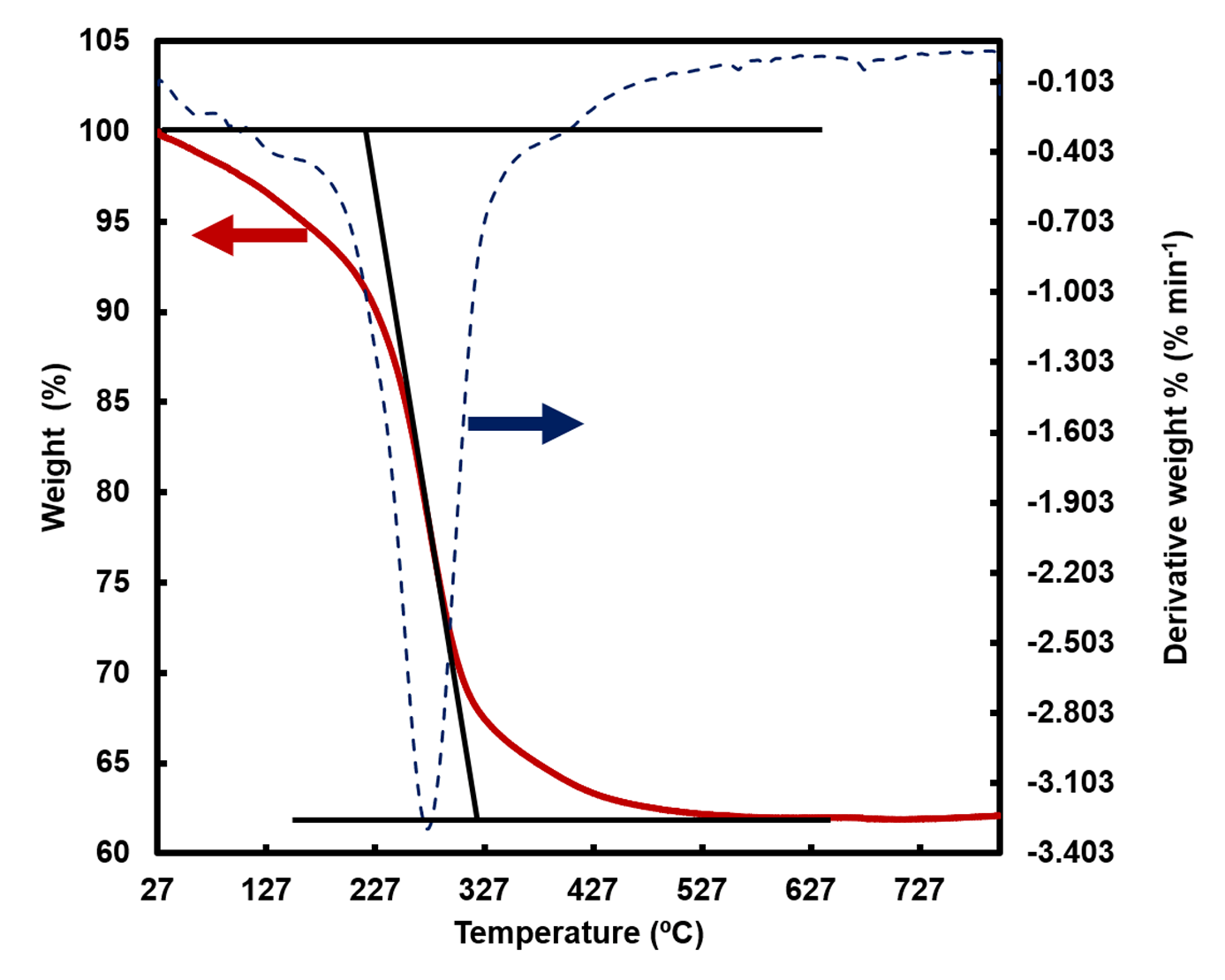
**

**Figure S1.** Thermogravimetric analysis of Hb@Ru NPs under nitrogen atmosphere. Heating rate: 10^o^C min^-1^.


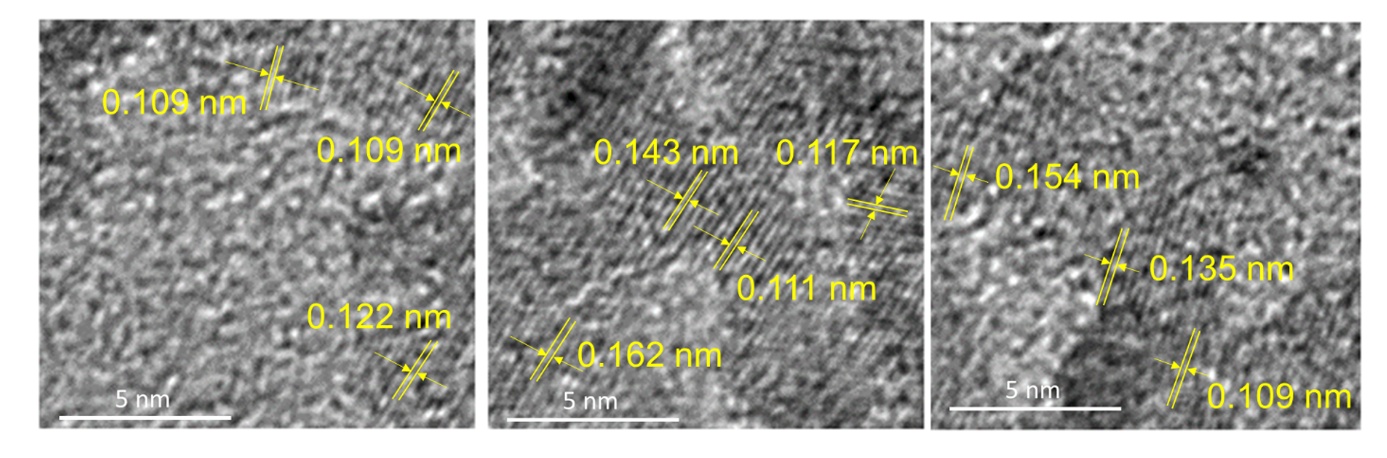


**Figure S2**. Zoomed-in HRTEM frames taken from different regions for exemplifying the d-spacing values within Hb@Ru NPs. The scale bars are given on the images.

**
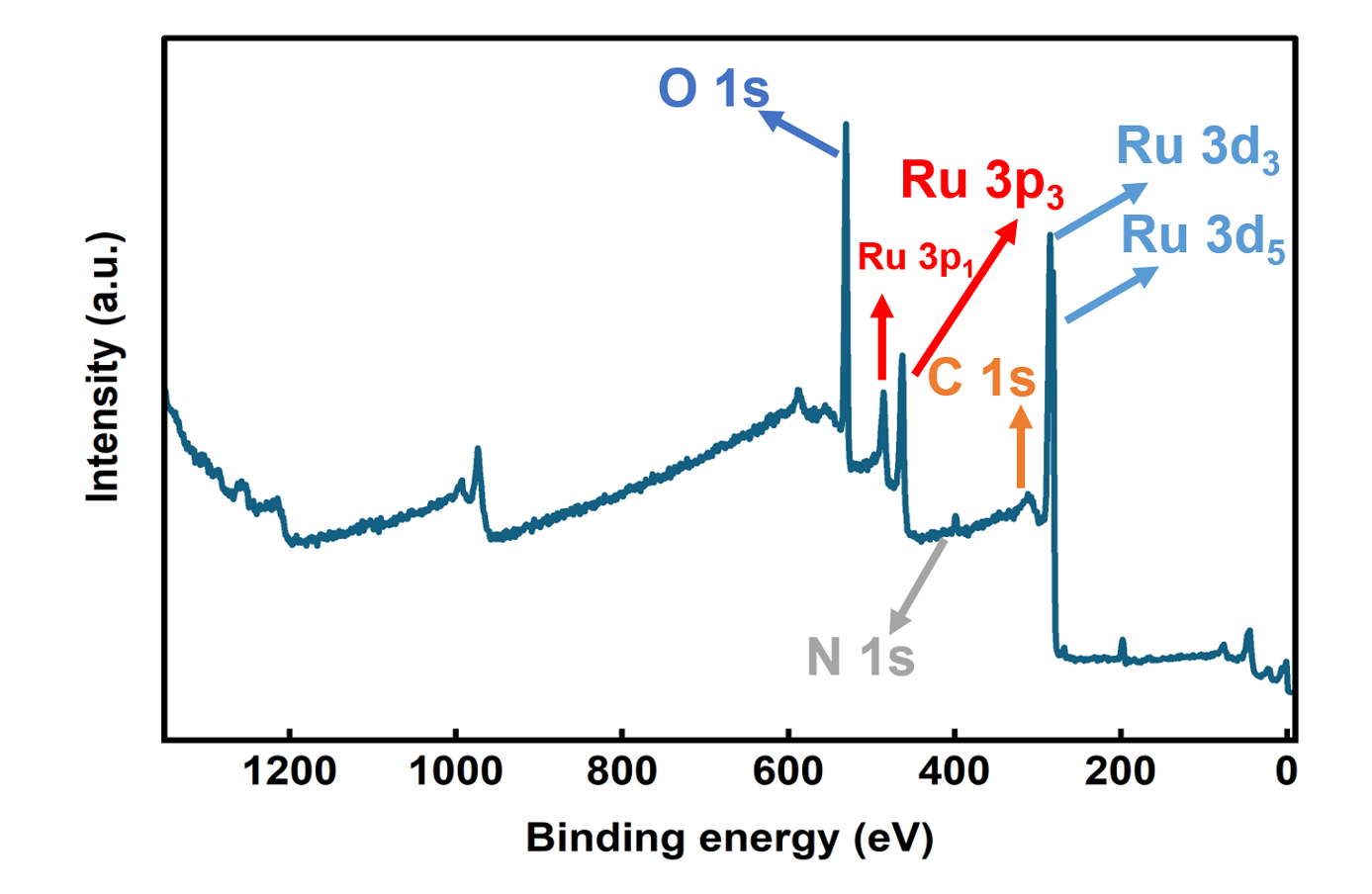
**

**Figure S3.** (A) Survey X-ray photoelectron spectroscopy of Hb@Ru NPs.

**Table S2**. X-ray photoelectron spectroscopy characterictics of Hb@Ru NPs.

| **Element** | **Core Level** | **Binding Energy (eV)** | | **Chemical State** | **Atomic %** |
| --- | --- | --- | --- | --- | --- |
| **Ru** | Ru 3d_5/2_ | | 281.8 | Ru^0^ | 20.1 |
|  |  |  | 282.9 | Ru^4+^ | 20.5 |
|  | Ru 3d_3/2_ | | 286.0 | Ru^0^ | 15.2 |
|  |  |  | 287.1 | Ru^4+^ | 14.2 |
|  | Ru 3p_1/2_ | | 463.1 | Ru^0^ | 27.1 |
|  |  |  | 464.3 | Ru^4+^ | 37.0 |
|  | Ru 3p_3/2_ | | 485.5 | Ru^0^ | 20.1 |
|  |  |  | 486.7 | Ru^4+^ | 15.9 |
| **C** | C 1s | | 284.8 | C-C | 15.2 |
|  |  |  | 288.7 | C=O | 10.4 |
| **O** | O 1s | | 529.5 | Ru-O-Ru | 27.3 |
|  |  |  | 530.8 | Ru-OH | 42 |
|  |  |  | 532.2 | -OH | 30.7 |
| **N** | N 1s | | 398.9 | C-N=C | 70.3 |
|  |  |  | 400.7 | C-N | 29.7 |

**S3. Catalase- and Peroxidase-like Activity of HbRu NPs.**


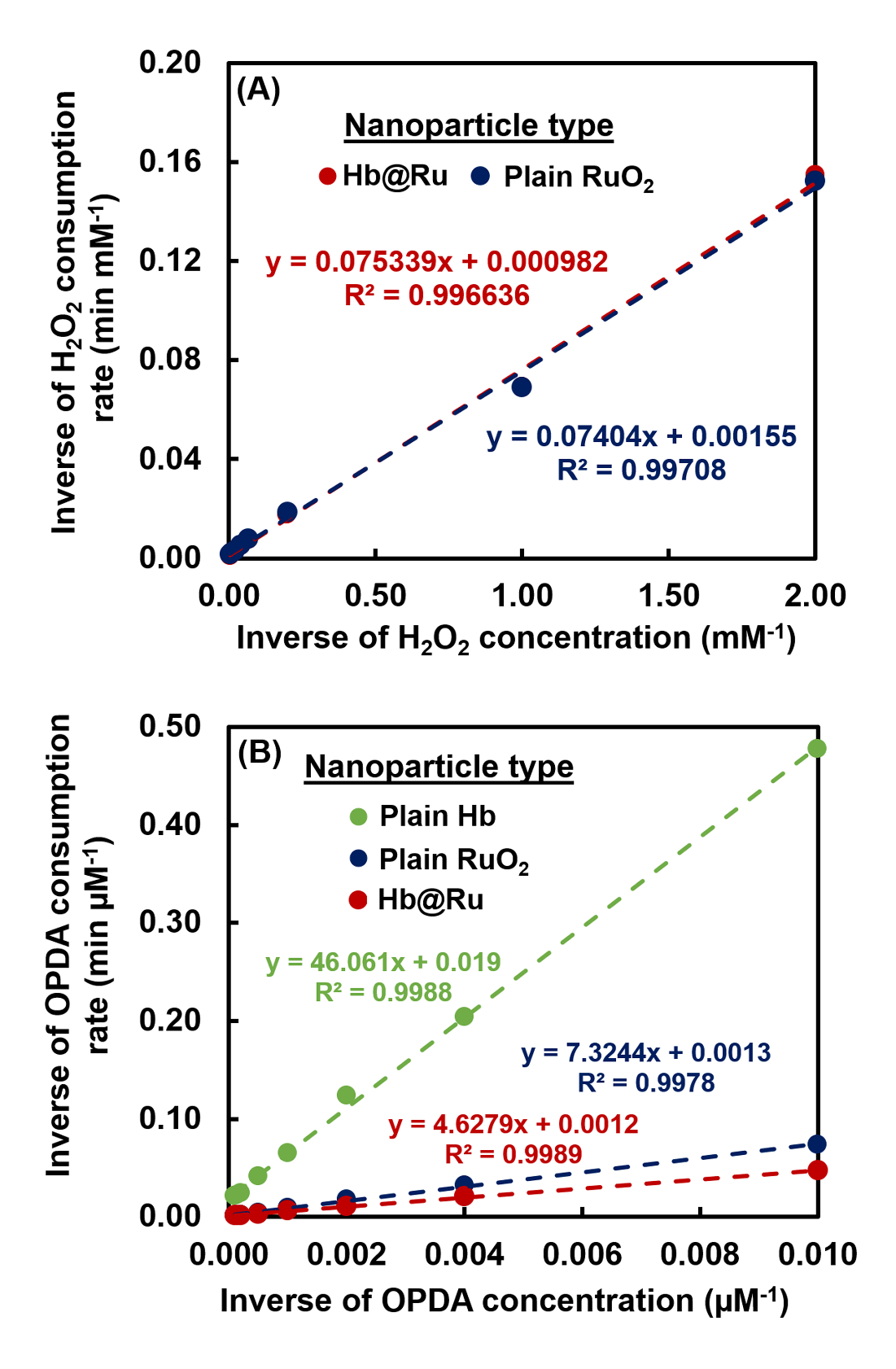


**Figure S4.** Lineweaver-Burk plots for (A) catalase-like and (B) peroxidase-like activities of plain Hb, plain RuO_2_ and Hb@Ru NPs. Nanozyme concentration: 0.05 mg mL^-1^ for catalase-like, 1.0 mg mL^-1^ for peroxidase-like activity. Medium: 1x PBS buffer at pH 7.4, Temperature: 37°C, Stirring rate: 300 rpm.

**
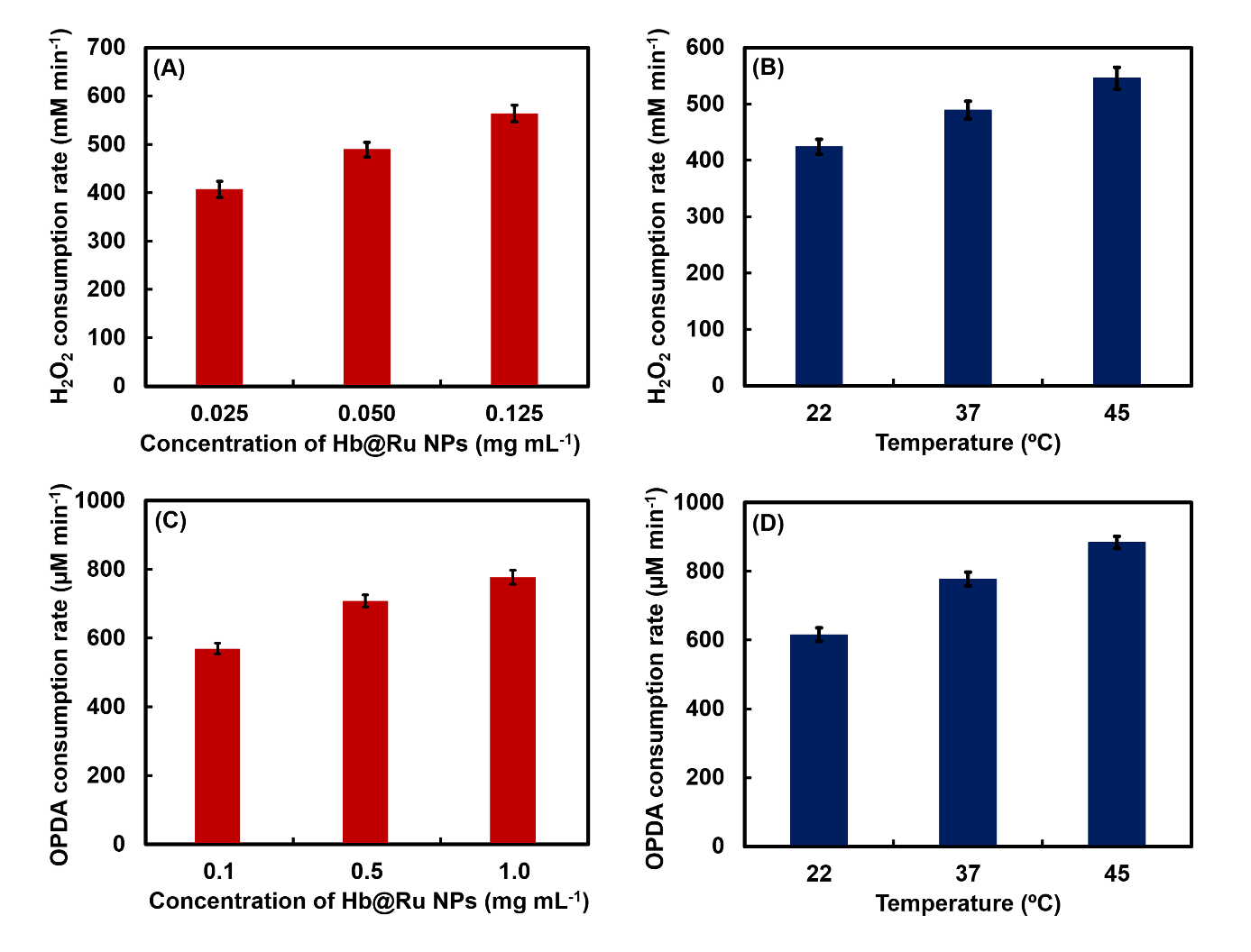
**

**Figure S5.** The effects of (A) nanozyme concentration and (B) temperature on catalase-like activity of Hb@Ru NPs. Unless otherwise stated, Nanozyme concentration: 0.05 mg mL^-1^. H_2_O_2_ concentration:100 mM. Medium: 1x PBS buffer at pH 7.4. Temperature: 37^o^C, Stirring rate: 300 rpm. The effects of (C) nanozyme concentration and (D) temperature on peroxidase-like activity of Hb@Ru NPs. Unless otherwise stated, Nanozyme concentration: 1.0 mg mL^-1^. 10 mM OPDA, H_2_O_2_ concentration: 0.5 mg mL^-1^. Medium: 1x PBS buffer at pH 7.4. Temperature: 37^o^C, Stirring rate: 300 rpm.

,

**S4. The effect of Hb@Ru NP concentration on GSH depletion behavior**

**
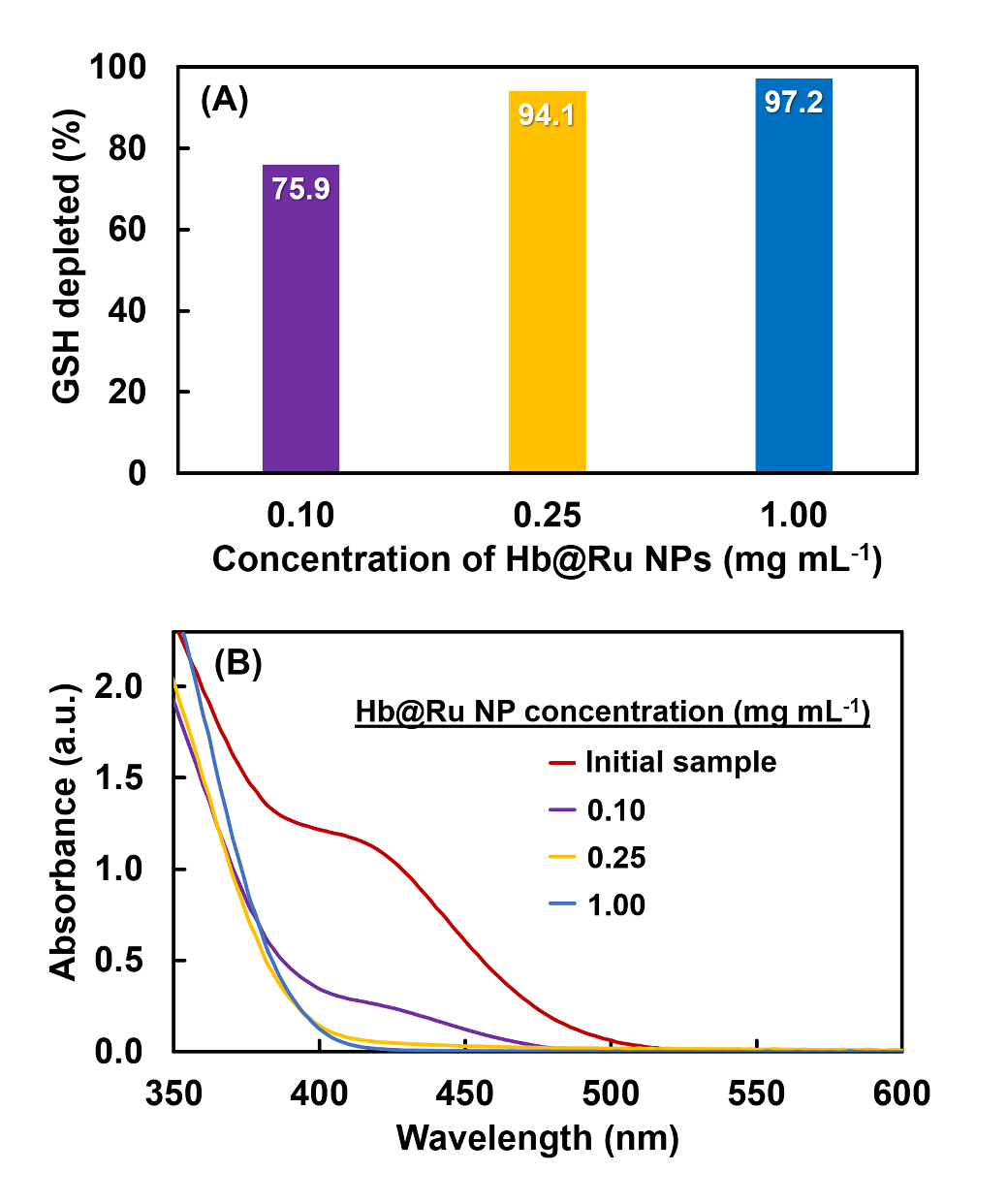
**

**Figure S6.** (A) The effect of Hb@Ru NP concentration on GSH depletion behavior. (A) The comparison of GSH depleted by Hb@Ru NPs at different concentrations. (B) Sample UV-Vis spectra recorded for GSH depletion with Hb@Ru NPs at different concentrations. Medium: 1x PBS buffer at pH 7.4, Temperature: 37^o^C. Initial GSH concentration: 0.5 mM. Time: 1 h.

**S5. Storage Stability of Hb@Ru NPs**

**
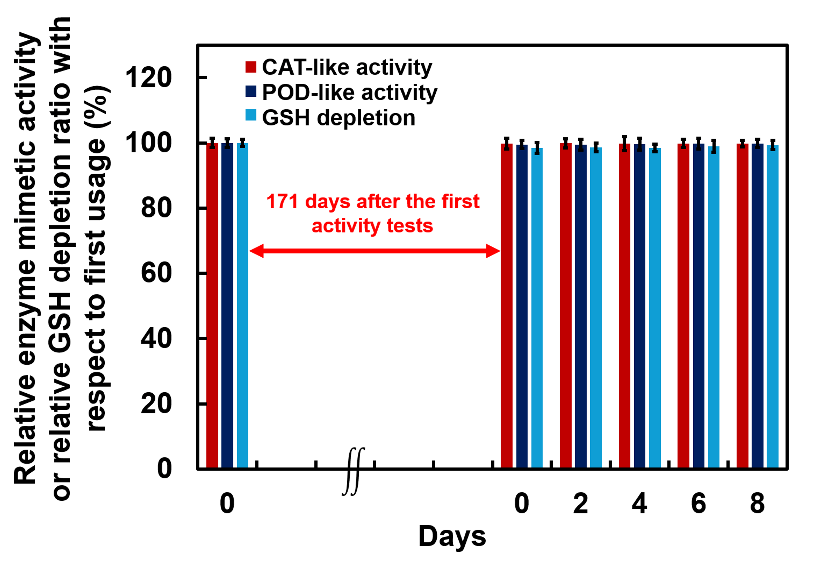
**

**Figure S7**. The storage stability of Hb@Ru NPs. The variation of enzyme-mimetic activities and GSH depletion behavior of Hb@Ru NPs with time. CAT-like activity conditions: Nanozyme concentration: 0.5 mg mL^-1^, DI water, 22^o^C, 300 rpm, POD-like activity conditions: Nanozyme concentration: 1.0 mg mL^-1^, 50 mM Tris buffer at pH 7.0, 22^o^C, 300 rpm. GSH depletion conditions. Initial GSH concentration: 0.5 mM, 50 mM phosphate buffer, pH: 7.0, 37^o^C, 300 rpm.

**S6. Nanomotor ability of Hb@Ru NPs**


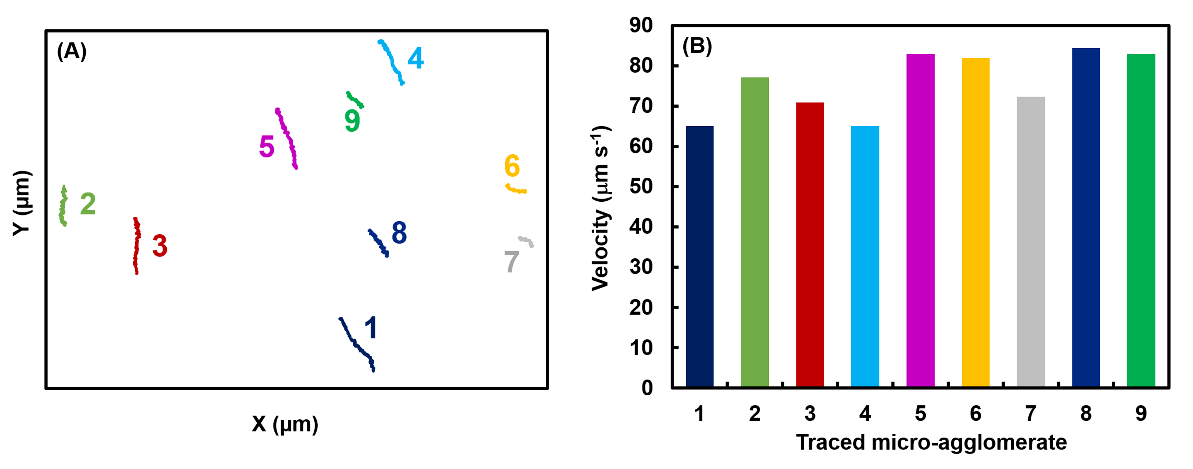


**Figure S8**. (A) Trajectory of the motion of monitored microagglomerates of Hb@Ru NPs on XY plane determined by the image analysis on a certain part of **Movie S2**. (B) The linear velocities of monitored micro-agglomerates. Medium: 1x PBS buffer at pH 7.4. H_2_O_2_ concentration: 250 mM.

**S7. Quantification of Hb@Ru NPs uptaken by cells in the absence of presence of H_2_O_2_**

The cultured T98G cells were removed by trpsinization for obtaining a cell density of 2x10^4^ cells mL^-1^ in a High glucose DMEM medium (1 mL). Hb@Ru NPs was added into the cell suspension at a concentration of 0.05 mg mL^-1^. The suspension was incubated for 30 min at 37^o^C in 5.0 % CO_2_ atmosphere for uptake Hb@Ru NPs by the cells. Concentrated H_2_O_2_ was added into the suspension at a concentration of 1.0 mM. Subsequently, the resulting suspension was further incubated for 5 min at 37^o^C in 5.0 % CO_2_ atmosphere. The cells was isolated from the suspension by centrifuging at 800 rpm for 5 min. The cells were resuspended in a PBS solution containing 2.0 % Triton-X (1.0 mL) and the resulting suspension was incubated for 10 min at 37^o^C in 5.0 % CO_2_ atmosphere for lysis of cells with uptaken Hb@Ru NPs. The cells were again removed from the suspension by centrifugation at 800 rpm for 5 min. The absorbance of the supernatant containing Hb@Ru NPs uptaken by lysed cells was measured in a micro-plate reader (Biochrom Ez Read 400, U.S.A.) at 570 nm.


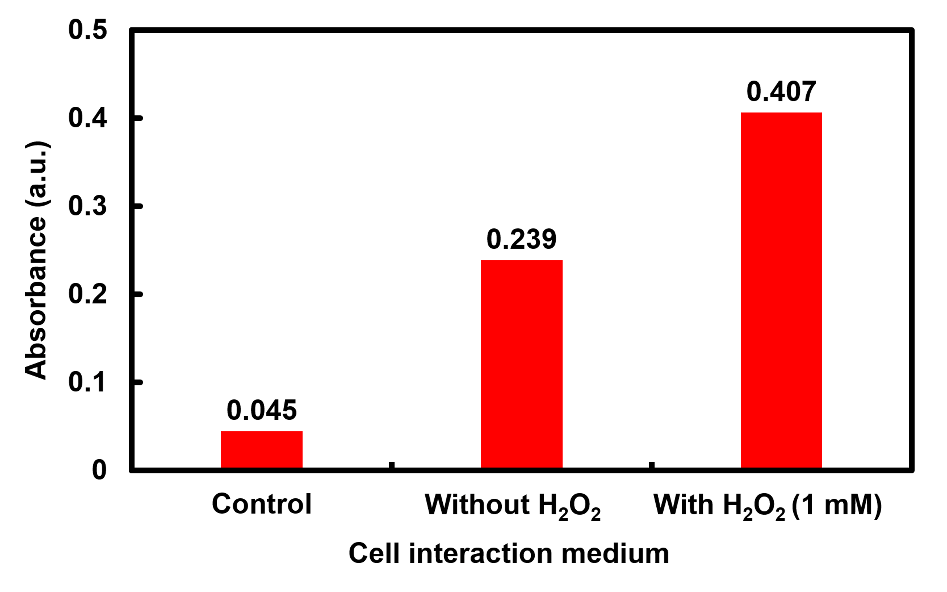


**Figure S9**. The comparison of the visible-region absorbance of PBS dispersion of Hb@Ru NPs uptaken by T98G cells in the absence of presence of H_2_O_2_ in High glucose DMEM medium. H_2_O_2_ concentration: 1 mM, T98G cell density: 2x10^4^ cells mL^-1^. Incubation period with H_2_O_2_: 5 min.

**S8. Fluorescence assay for testing the generation of •OH radicals by plain Hb, Plian Ru and Hb@Ru NPs**


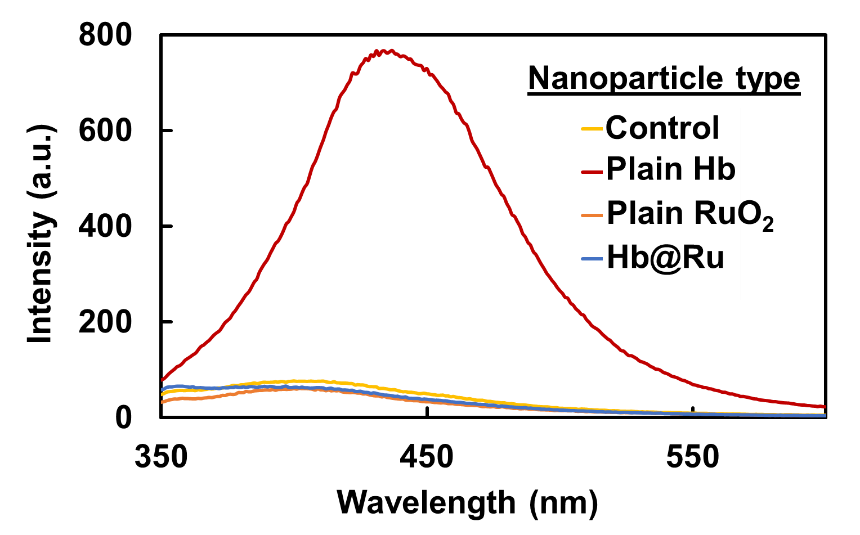


**Figure S10**. (A) Fluorescence spectra obtained with plain Hb, plain RuO_2_ and Hb@Ru NPs for observing ^•^OH radical generation via the formation of 2-HTPA as the fluorescent probe. Excitation: 315 nm, emission: 430 nm, Concentration of plain Hb, plain RuO_2_ and Hb@Ru NPs: 1.0 mg mL^-1^. H_2_O_2_ concentration: 5 mM, 22^o^C.

**S9. Processing of Intracellular ROS images**


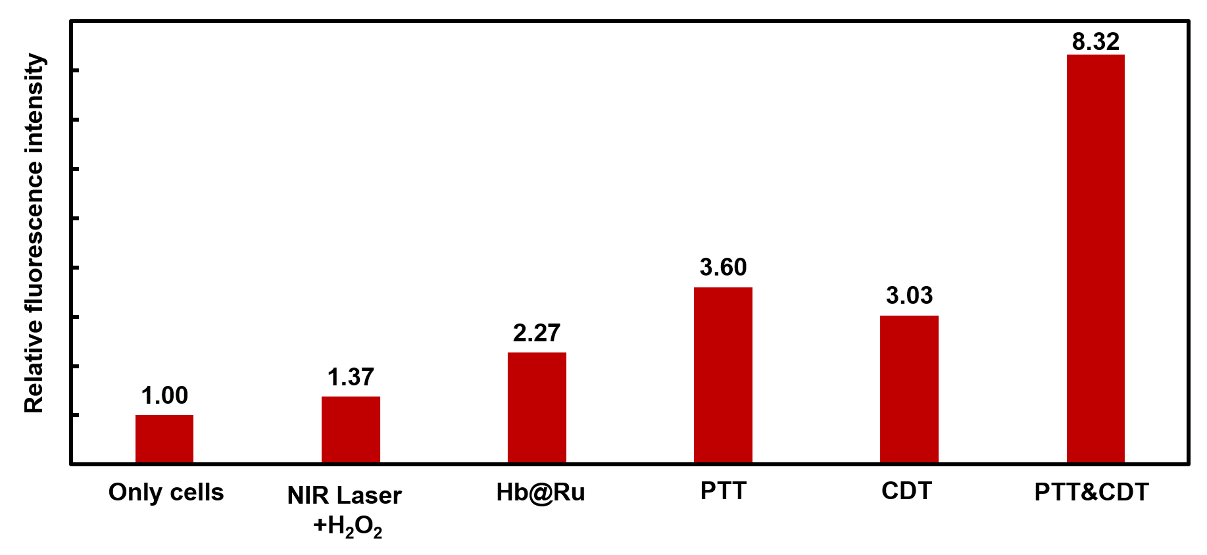


**Figure S11**. The plot showing the change of relative fluoresce intensity in the intracellular ROS experiments performed with different therapeutic modalities. The relative fluorescence intensity was defined as the ratio of fluorescence intensity determined on a selected frame via Image J® to the fluorescence intensity observed with only cells.

**S10. Photothermal Properties of Hb@Ru NPs.**


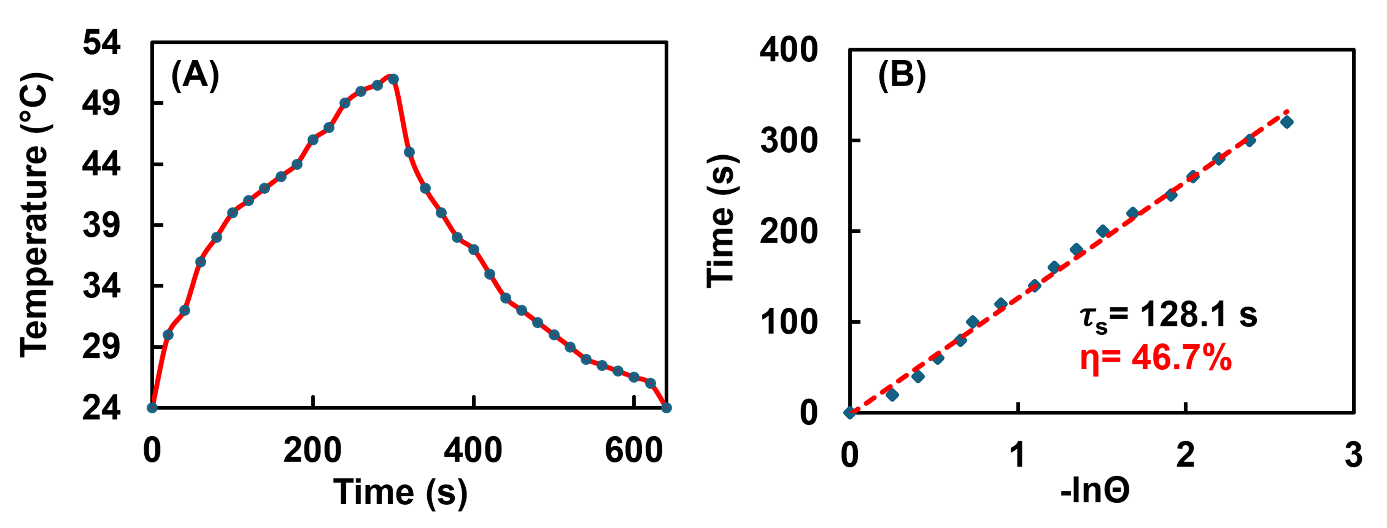


**Figure S12.** (A) A typical successive heating/cooling curve with Hb@Ru NPs at a concentration of 1.0 mg mL^-1^. (B) The determination of photothermal efficiency (η) of Hb@Ru NPs based on time constant method.

**
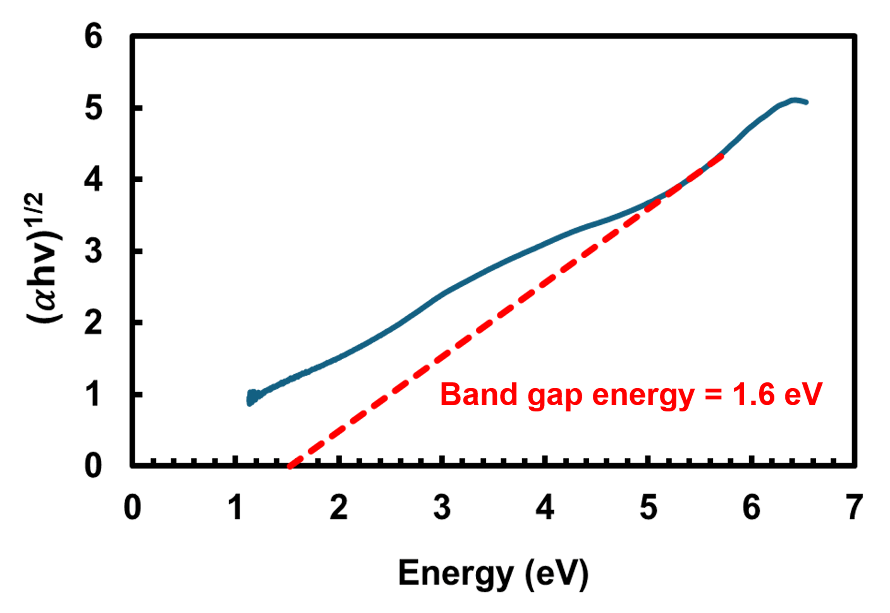
**

**Figure S13.** The Tauc plot sketched for the determination of band gap energy for Hb@Ru NPs.


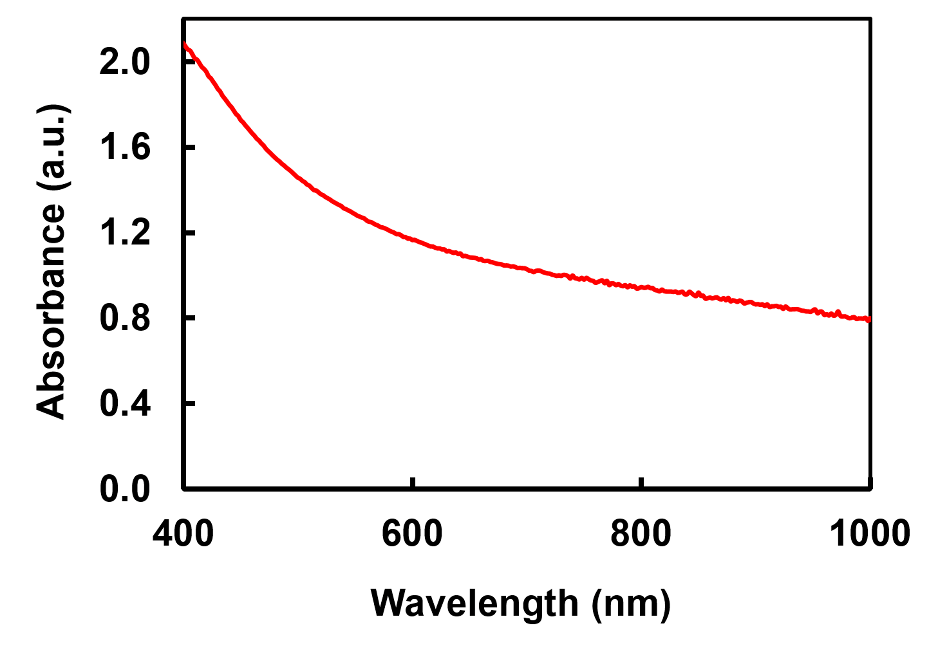


**Figure S14**. The absorption spectrum of Hb@Ru NPs recorded to demonstrate the appreciable light absorption in NIR-I region. Concentration of Hb@Ru NPs: 0.125 mg mL^-1^.

**S11. Cytotoxicity of Hb@Ru NPs**


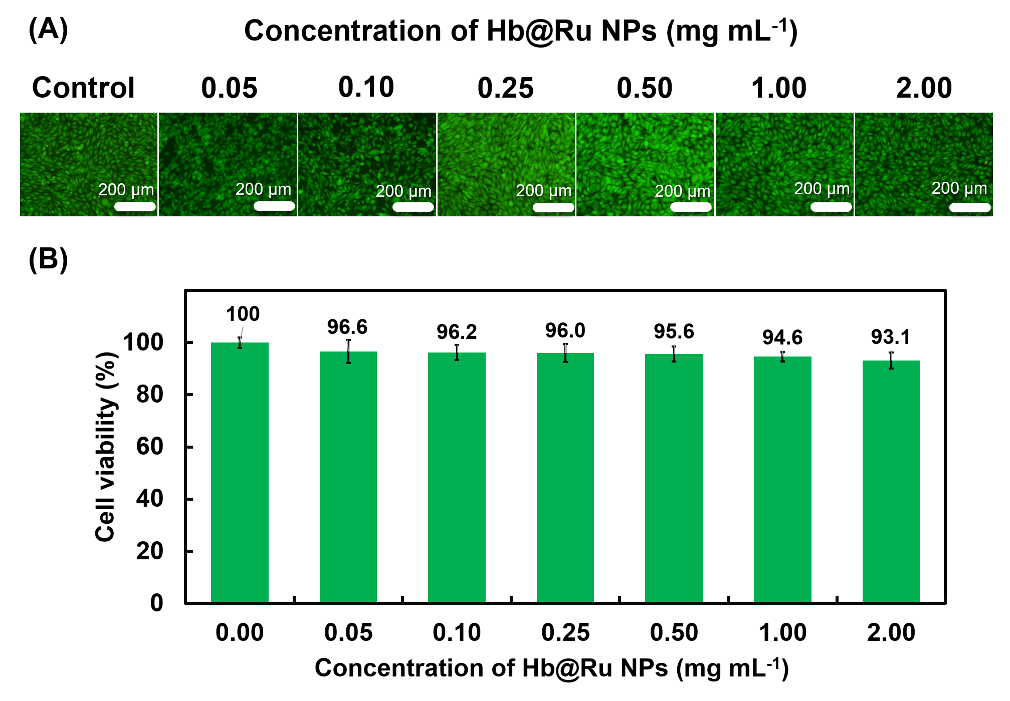


**Figure S15.** The variation of viability of L929 cells with the concentration of Hb@Ru NPs. (A) Live/dead L929 cell images taken after dual cell staining with AO/PI system, after interaction with Hb@Ru NPs at different concentrations. Scale bar: 200 µm, L929 cell density: 2x10^4^ cells well^-1^. (B) MTT test results showing the viability of L929 cells following interaction with Hb@Ru NPs at different concentrations. The control image was obtained in the absence of Hb@Ru NPs.

**
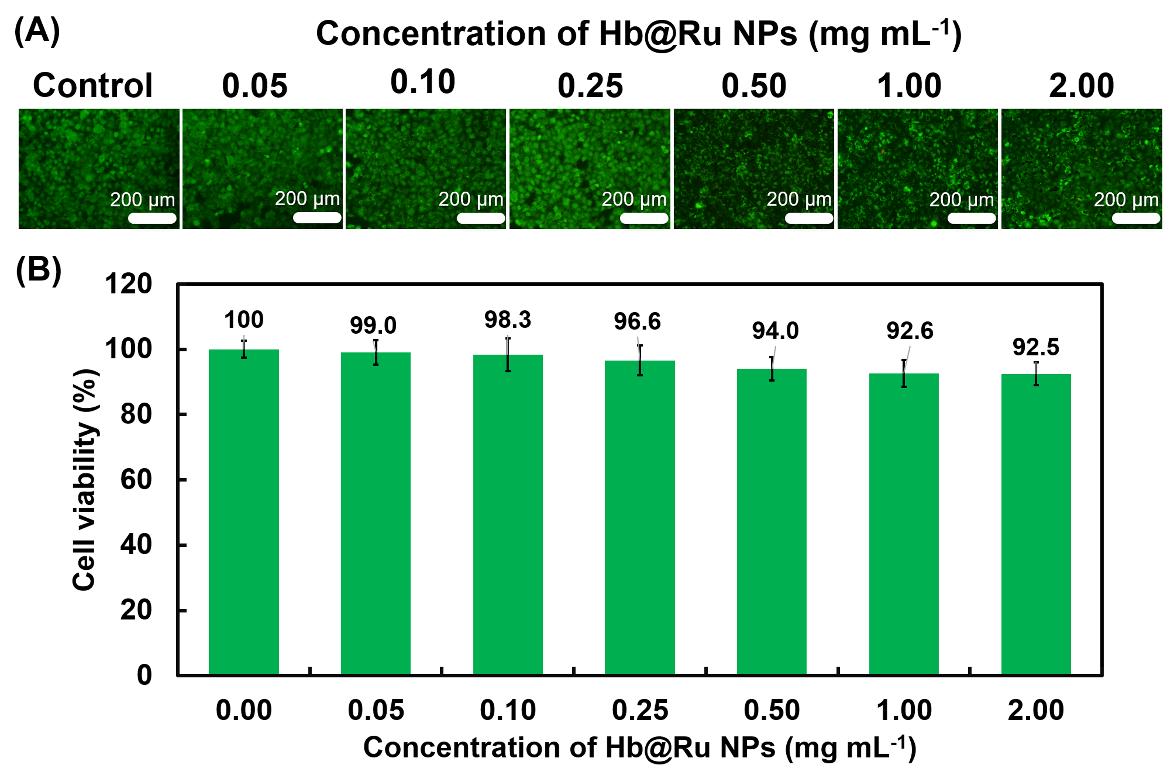
**

**Figure S16. (**A) Live/dead T98G cell images taken after dual cell staining with AO/PI system, after interaction with Hb@Ru NPs at different concentrations. Scale bar: 200 µm, T98G cell density: 2x10^4^ cells well^-1^. (B) MTT test results showing the viability of L929 cells following interaction with Hb@Ru NPs at different concentrations. The control image was obtained in the absence of Hb@Ru NPs.

**S12. PTT&CDT Modality with HepG2 cells**

**
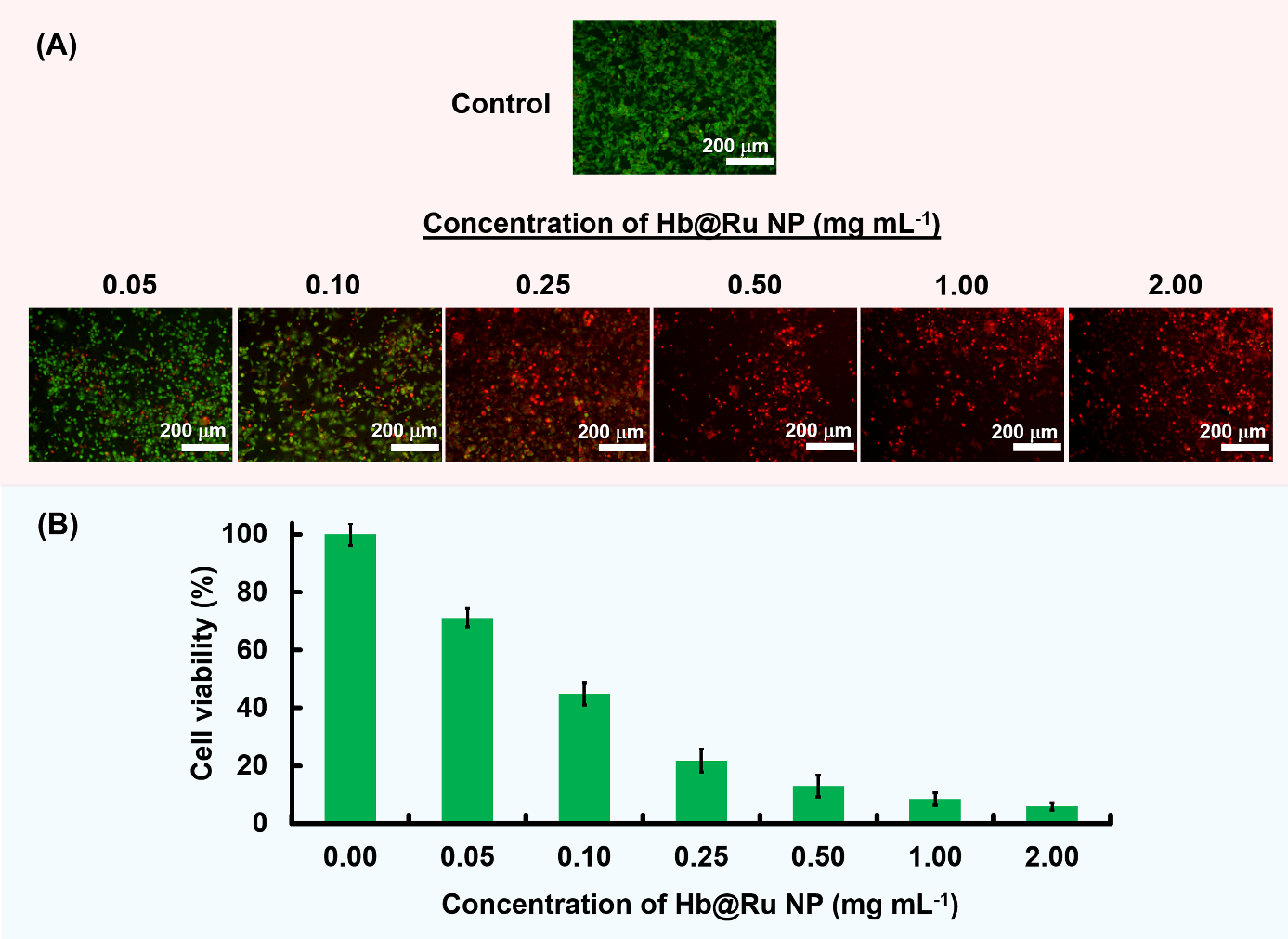
**

**Figure S17**. (A) The control image obtained with HepG2 cells and representative Live/dead HepG2 cell images obtained with PTT&CDT modality with different concentrations of Hb@Ru NPs, PTT&CDT combination was applied using NIR laser at 808 nm with a power density of 1 W cm^-2^ in the presence of 1 mM exogeneous H_2_O_2_ for 5 min. Scale bar: 200 μm. (B): MTT results demonstrating the viability of HepG2 cells after interaction with Hb@Ru NPs at different concentrations in PTT&CDT modality. Number of replicates: 3. Mean±SD.

**S13. Stratch Assay Plot**

**
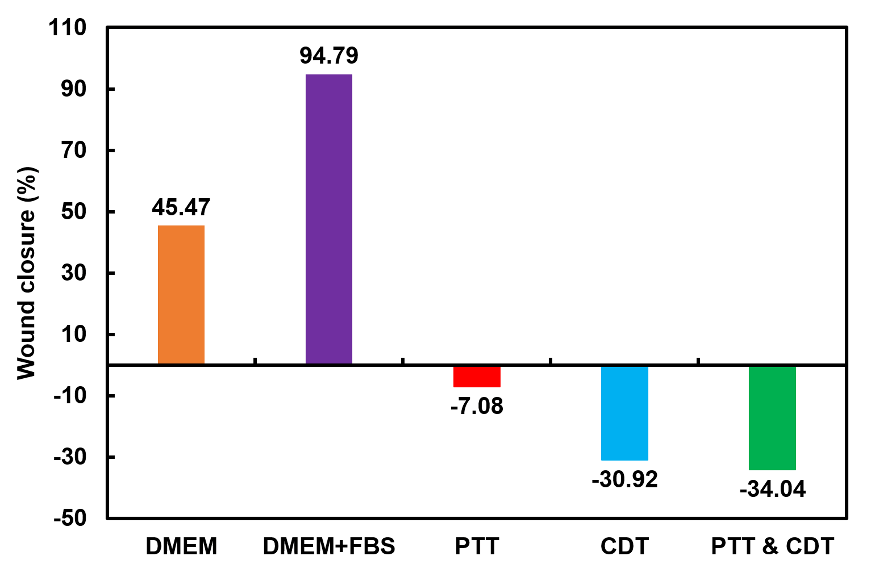
**

**Figure S18**. The wound closure plot obtained by the comparative usage of Image J® wound healing size tool for the images taken initially and at 48 h in **Figure 8C**.


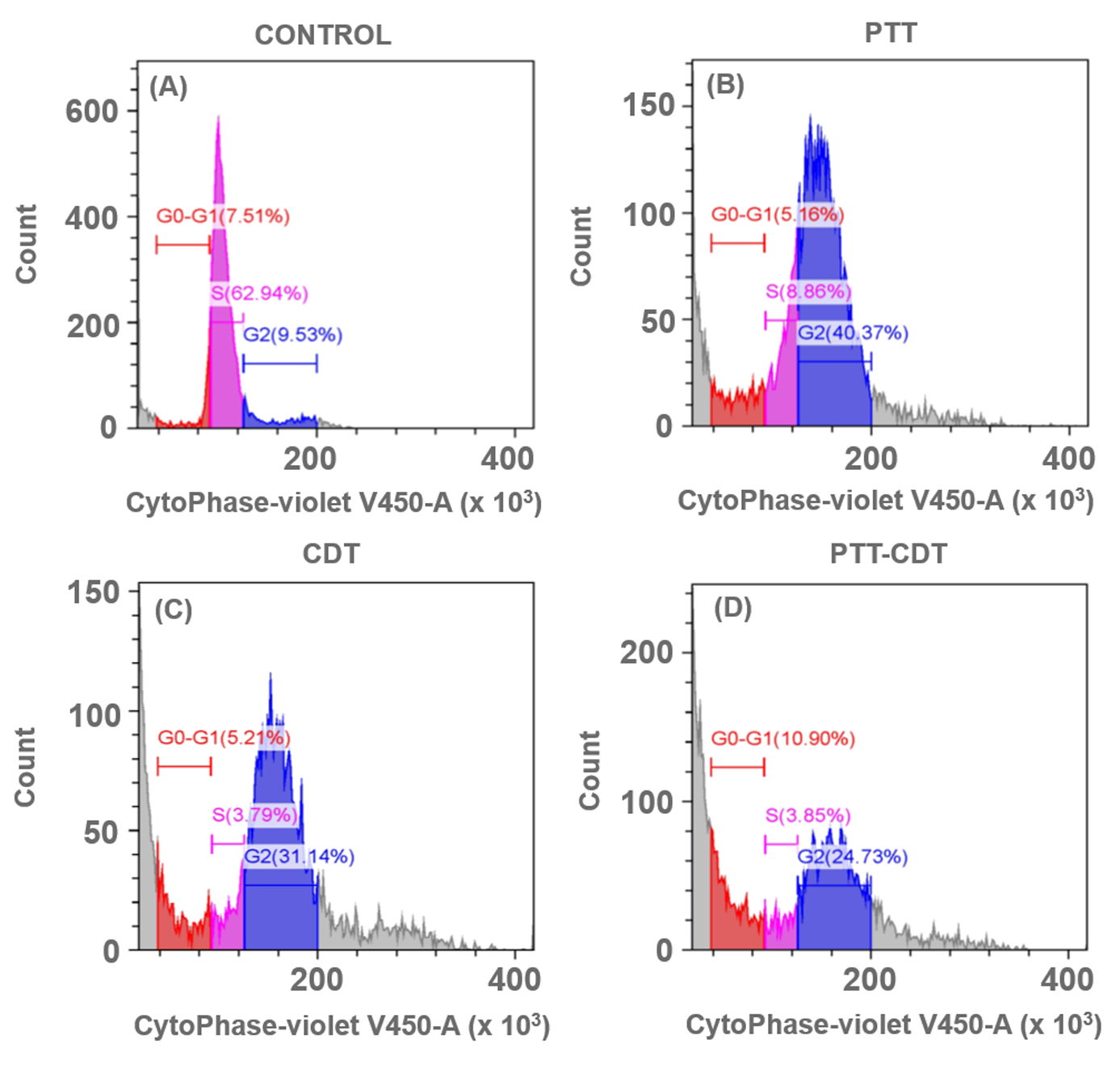


**Figure S19**. Flow cytometric analysis of cell cycle distribution in T98G cells following different therapeutic treatments. Cells were stained with CytoPhase™ Violet and analyzed using a CytoFLEX LX Flow Cytometer. (A) Control, (B) CDT, (C) PTT, and (D) PTT&CDT groups showing the distribution of cells in Sub-G1, G0/G1, S, and G2/M phases.

**References**

S1. Sungu Akdogan C-Z, Akbay Cetin E, Onur M-A et al (2025) Copper(II) oxide spindle-like nanomotors decorated with calcium peroxide nanoshell as a new nanozyme with photothermal and chemodynamic functions providing ros self-amplification, glutathione depletion, and Cu(I)/Cu(II) Recycling. ACS Appl Mater Interfaces 17:632-649. <https://doi.org/10.1021/acsami.4c17852>.

S2. Kip Ç, Akbay Çetin E, Gökçal Kapucu B et al (2025) Ultrafine palladium based nanozyme exhibiting photothermal and chemodynamic responses with O_2_ bubble driven motion and glutathione depletion ability. J Drug Deliv Sci Technol 106:106728. <https://doi.org/10.1016/j.jddst.2025.106728>.

S3. Hacıibrahimoğlu S, Akbay Çetin E, Gökçal Kapucu B et al (2026) A hollow mesoporous manganese oxide based multifunctional nanozyme with self-propelled nanomotor behavior and direct copper binding ability for synergistic therapy of glioblastoma with hypoxia alleviation. Mater Today Nano 33:100738. <https://doi.org/10.1016/j.mtnano.2025.100738>.

S4. Sungu Akdogan C-Z, Akbay Cetin E, Onur M-A et al (2024) In vitro synergistic photodynamic, photothermal, chemodynamic, and starvation therapy performance of Chlorin e6 immobilized, polydopamine-coated hollow, porous ceria-based, hypoxia-tolerant nanozymes carrying a cascade system. ACS Appl Bio Mater 7: 2781-2793. <https://doi.org/10.1021/acsabm.3c01181>.

S5. Sungu Akdogan C-Z, Gokcal B, Polat M et al (2022) Porous, oxygen vacancy enhanced CeO_2-x_ microspheres with efficient enzyme-mimetic and photothermal properties. ACS Sustain Chem Eng 10: 9492-9505. <https://doi.org/10.1021/acssuschemeng.2c01981>.

S6. Morais E, Thampi K-R, Sullivan J-A (2020) Photo-Dissociation of CO_2_ over Plasmonic RuO_2_ Nanoparticles. ChemistrySelect 5: 3069-307, <https://doi.org/10.1002/slct.202000161>.
